# Cell competition drives bronchiolization and pulmonary fibrosis

**DOI:** 10.21203/rs.3.rs-4177351/v1

**Published:** 2024-04-22

**Authors:** Rachel Warren, Kylie Klinkhammer, Handeng Lyu, Changfu Yao, Barry Stripp, Stijn P. De Langhe

**Affiliations:** 1 Department of Medicine, Division of Pulmonary and Critical Medicine, Mayo Clinic, Rochester, MN 55905, USA.; 2 Women’s Guild Lung Institute, Department of Medicine, Cedars-Sinai Medical Center, Los Angeles, CA 90048, USA

## Abstract

Idiopathic pulmonary fibrosis (IPF) is a progressive scarring disease arising from the maladaptive differentiation of lung stem cells into bronchial epithelial cells rather than into alveolar type 1 (AT1) cells, which are responsible for gas exchange. Here, we report that healthy lungs maintain their stem cells through tonic Hippo and β-catenin signaling, which promote Yap/Taz degradation and allow for low level expression of the Wnt target gene *Myc*. Inactivation of upstream activators of the Hippo pathway in lung stem cells inhibits this tonic β-catenin signaling and *Myc* expression and promotes their Taz mediated differentiation into AT1 cells. Vice versa, increased Myc in collaboration with Yap promotes the differentiation of lung stem cells along the basal and myoepithelial like lineages allowing them to invade and bronchiolize the lung parenchyma in a process reminiscent of submucosal gland development. Our findings indicate that stem cells exhibiting the highest Myc levels become supercompetitors that drive remodeling, whereas loser cells with lower Myc levels terminally differentiate into AT1 cells.

## Introduction

Idiopathic pulmonary fibrosis (IPF) pathogenesis encompasses alveolar and fibrotic remodeling, inflammation, and eventual loss of lung architecture^1^ resulting in progressive loss of pulmonary function, respiratory failure, and death often within 5 years of diagnosis^2,3^. Accumulating genetic data implicate impaired epithelial maintenance and function as drivers of pulmonary fibrosis^4–8^.

The alveolar epithelium is primarily comprised of alveolar type 2 stem cells (AT2s) and alveolar type 1 cells (AT1s) responsible for gas exchange. Club stem cells, located at the bronchio-alveolar duct junctions (bronchioalveolar stem cells (BASCs)), and AT2 stem cells are capable of self-renewal and differentiation into AT2 and/or AT1 cells through a pre-ATI transitional cell state (PATS) that has only recently been appreciated^9–11^. Hallmarks of ineffectual repair include the aberrant accumulation of PATS^9,10,12^ and ectopic airway differentiation, called bronchiolization, a prominent feature of interstitial lung disease^12–16^. In vivo, there is no evidence for AT2 stem cells and some evidence for Club cells contributing to bronchiolization. In fact, upon H1N1 influenza injury the stem cells driving this bronchiolization have been demonstrated to be intralobular serous cells^17^, intralobular airway-resident basal p63+ progenitors^18^ and preexisting basal cells (BCs)^19^ all of which depend on Trp63. For the purpose of this manuscript, we will group these together as basal-like cells (BLCs). Once established bronchiolization is difficult to resolve and this persistence of bronchial epithelial cells incapable of gas exchange ultimately leads to death. However, genetic interventions have suggested that it may be possible to reprogram these bronchiolized areas into alveolar epithelium and potentially cure this disease^20,21^.

Interestingly, whether bronchiolization occurs seems to largely depend on the level of injury, e.g. catastrophic injury to the lung parenchyma which wipes out the majority of AT2s, AT1s and Club cells, suggesting that some form of cell competition may be at play. Indeed, BLCs are resistant to influenza A virus^21^ and SARS-Cov2^22^ infection. Therefore, one possibility is that BLCs under normal conditions are kept at bay by “more competitive” Club cells or AT2 stem cells. Interestingly, upon Sendai virus infection which only destroys Club cells and AT2 cells but not AT1 cells^23^, BLCs have been shown to outcompete and replace surviving AT1 cells and bronchiolize the lung parenchymal regions devoid of AT2 stem cells^24^.

In tissues harboring a mosaic imbalance in Myc protein levels, cells with higher Myc levels expand at the expense of cells with lower levels by eliminating them through apoptosis, inducing senescence, promoting autophagy or directing them to terminal differentiation and sloughing^25^. Cells measure their Myc content relative to their neighbors, and cells with lower Myc levels are eliminated by neighbors with higher Myc^26,27^. This process is known as cell competition^25,28–32^. Cells that grow faster and eliminate less-fit cells are called super-competitors. Cells become super-competitors when their levels of Myc expression are two-fold higher than that of their neighbors^26,28,33,34^. This process may reflect a selection for fit cells, since Myc maintains stemness, eliminating cells with lower Myc may guard against premature differentiation.

Here, we demonstrate that lung epithelial stem cell competitiveness/fitness levels are determined by their Myc levels, which are tuned by the Hippo pathway. Healthy lungs maintain their stem cells through tonic Hippo and β-catenin signaling, which promote Yap/Taz degradation and allow for low level expression of the Wnt target gene *Myc*. Inactivation of upstream activators of the Hippo pathway in AT2 or Club stem cells stabilizes Yap/Taz, which inhibit Myc expression by promoting β-catenin degradation and allows for Taz to translocate to the nucleus and drive AT1 cell differentiation. Vice versa, increased Myc in collaboration with Yap promotes the differentiation of lung stem cells along the basal and myoepithelial like lineages allowing them to invade and bronchiolize the lung parenchyma in a process reminiscent of submucosal gland development. Our findings indicate that stem cells exhibiting the highest Myc levels become supercompetitors that drive remodeling, whereas loser cells with lower Myc levels terminally differentiate into AT1 cells.

## Results

### Club cells compete with BLCs to regenerate vs bronchiolize the lung parenchyma upon catastrophic injury to the lung parenchyma.

After catastrophic injury to the lung parenchyma by influenza infection, bronchial epithelial stem cells (BESCs) in the airway have been proposed to undergo a binary response to reconstitute epithelial barriers giving rise to either alveolar epithelium or to generate more airway epithelium and “bronchiolize” the lung parenchyma. However, it has been unclear whether one particular BESC subpopulation undergoes this binary response or whether there is competition between different BESC populations capable of either promoting alveolar epithelial regeneration or bronchiolization. This is largely because most lineage tracing experiments to target BESCs rely on *Sox2*^*CreERT2*^*;mTmG* mice which lineage labels all bronchial epithelial cells.

**Figure 1.**
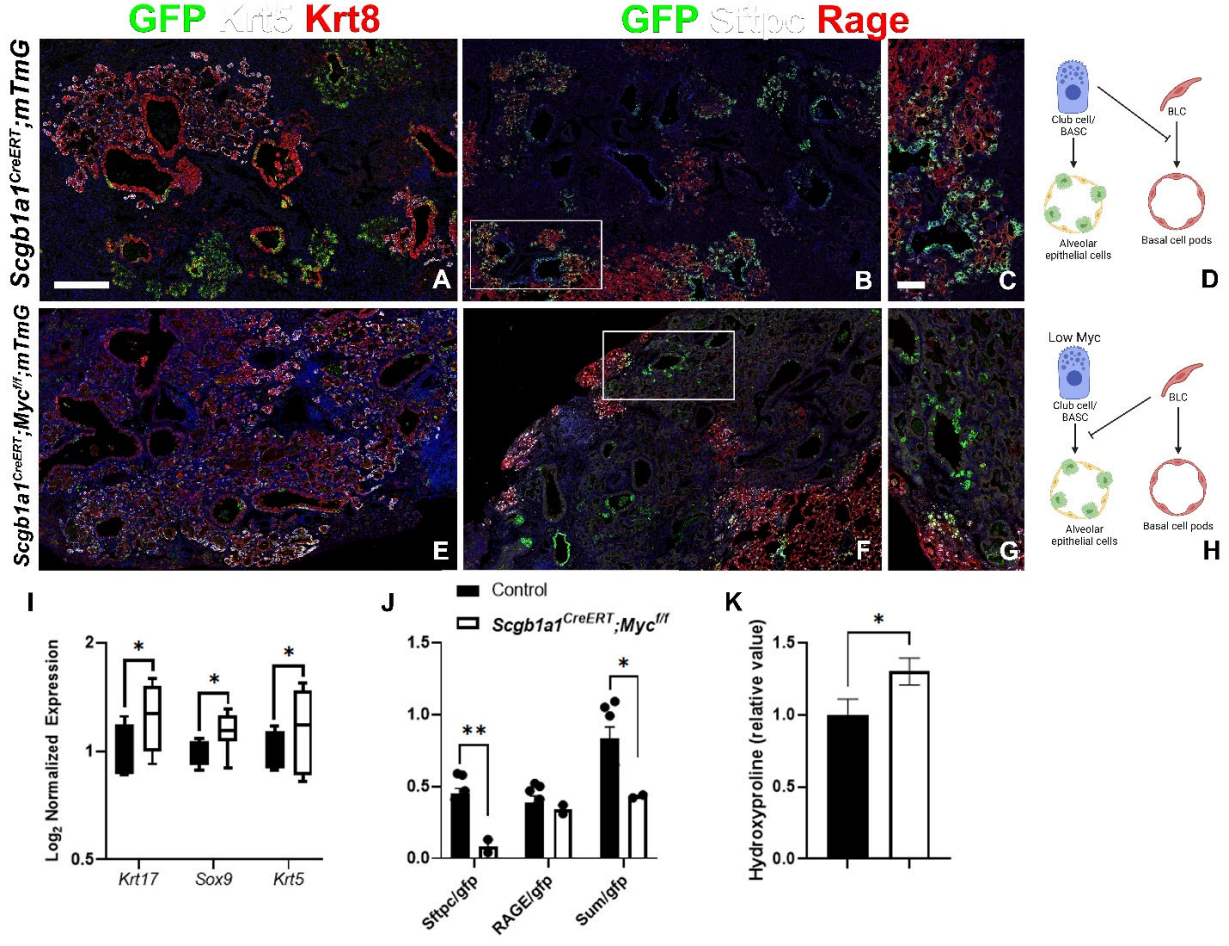
Club cells and bronchoalveolar stem cells require Myc to inhibit basal-like cells and promote regeneration following influenza injury. *Scgb1a1*^*CreERT*^*;mTmG* and *Scgb1a1*^*CreERT*^*;Myc*^*f/f*^*;mTmG* were placed on tamoxifen containing chow at 8 weeks of age for 3 weeks to inactivate Myc and permanently label all Club cells/BASCs and their offspring with GFP. After a 3 week wash-out period, mice were intranasally administered 13,000 viral foci units (VFU) of H1N1 influenza virus. At 6 weeks post injury, left lung lobes were inflation fixed, embedded in paraffin, and sectioned. (A, E) Coimmunostaining for GFP (lineage label), Keratin5 (Krt5; basal and basal-like cells), and Keratin 8 (Krt8; basal-like cells and transitional cells) on *Scgb1a1*^*CreERT*^*;mTmG* and *Scgb1a1*^*CreERT*^*;Myc*^*f/f*^*;mTmG* lung sections. (B, C, F, G) Coimmunostaining for GFP (lineage label), surfactant protein C (Sftpc; AT2 cells), Rage (AT1 cells) on *Scgb1a1*^*CreERT*^*;mTmG* and *Scgb1a1*^*CreERT*^*;Myc*^*f/f*^*;mTmG* lung sections. White boxes in B, F are depicted in C, G, respectively. (D) Model demonstrating that Myc sufficient Club cells inhibit basal cells and give rise to alveolar epithelial cells. (H) Model demonstrating that Myc deficient Club cells and alveolar regeneration are inhibited by Myc sufficient basal cells. (I) Nanostring nCounter analysis on RNA from *Scgb1a1*^*CreERT*^*;mTmG* (N=4) and *Scgb1a1*^*CreERT*^*;Myc*^*f/f*^*;mTmG* (N=7) lungs for BLC genes including *Keratin 17, Sox9*, and *Krt5*. Data are Log2 normalized and graphed as a box and whiskers plot. (J) Lineage tracing analysis on immunostaining in B (N=7), E (N=2). Area of GFP, Sftpc, and Rage were determined using Aivia artificial intelligence software. Graph depicts the ratio of total area of Sftpc of the total GFP area, ratio of total Rage area of the total GFP area, and the ratio of the sum of Sftpc and Rage area of the total GFP area. (K) Hydroxyproline analysis for soluble collagen in *Cre-* controls and *Scgb1a1*^*CreERT*^*;Myc*^*f/f*^*;mTmG* lungs normalized to *Scgb1a1*^*CreERT*^*;mTmG.* Scale bar: 250μm. Student’s T test was used to determine significance. * p<0.05.

It is well known that subsets of Club cells (e.g. BASCs) can give rise to both airway and alveolar epithelium^35–37^ but do not contribute in a significant way to bronchiolization of the lung parenchyma after catastrophic injury mediated by H1N1 influenza^18^. However, because Club cells and especially BASCs are also destroyed by H1N1 influenza it has been difficult to assert whether they can contribute to alveolar epithelial regeneration if they survive the initial injury. To investigate this we used *Scgb1a1*^*CreER*^*;mTmG* mice to lineage label Club cells, including BASCs and performed H1N1 mediated injury. Our experiments confirm previous reports that Club cells do not participate in the bronchiolization of the lung parenchyma after H1N1 mediated injury (Fig1.A–D) which is known to be mediated by BLCs under this condition^18^. However, we find that if Club cells survive the initial assault they can contribute to alveolar epithelial regeneration after injury (Fig1.A–D). Remarkable, Club cells regenerating alveolar epithelium or BLCs bronchiolizing the lung parenchyma are mutually exclusive events, suggesting that when Club cells survive the initial assault, they can compete with BLCs, preventing them from entering and bronchiolizing the lung parenchyma.

In tissues harboring a mosaic imbalance in Myc protein levels, cells with higher Myc levels expand at the expense of cells with lower levels by eliminating them through apoptosis, inducing senescence, promoting autophagy or directing them to terminal differentiation and sloughing^25^. To investigate whether Club cells compete with BLCs using the classic cell competition model we inactivated *Myc* in Club cells specifically using *Scgb1a1*^*CreER*^*;Myc*^*f/f*^*;mTmG* mice while simultaneously lineage tracing them. We find that upon H1N1 injury alveolar epithelial regeneration by Club cells in *Scgb1a1*^*CreER*^*;Myc*^*f/f*^*;mTmG* mice is impaired with the majority being outcompeted by BLCs and the remainder giving rise to AT1 rather than AT2 cells compared to *Scgb1a1*^*CreER*^*;mTmG* control mice, that feature normal Myc levels. *Scgb1a1*^*CreER*^*;Myc*^*f/f*^*;mTmG* lungs featured increased bronchiolization mediated by BLCs and increased pulmonary fibrosis as measured by hydroxyproline content (Fig.1E–K). Together these findings indicate that stem cell competition in the lung is governed by Myc levels.

### Subsets of bronchial epithelial stem cells acquire myoepithelial cell characteristics after injury to the lung parenchyma.

**Figure 2.**
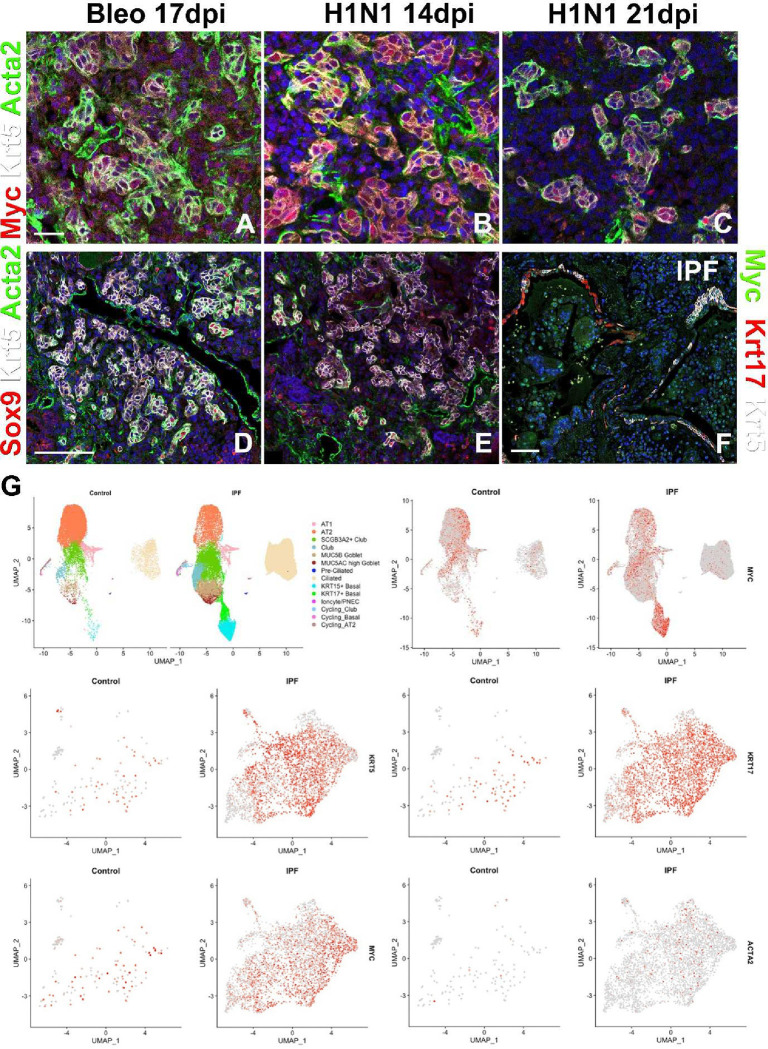
High expression of *Myc* in BLCs in IPF. (A-E) Coimmunostaining for myoepithelial cell markers Myc (A-C) or Sox9 (D,E), Krt5 and Acta2 (smooth muscle actin) on honeycomb regions in mouse lungs 17 days after bleomycin (A,D) and 14 or 21 days after H1N1 injury (B,C,E). (F) Coimmunostaining for Myc, Krt17, and Krt5 on honeycomb regions in human IPF tissue. (G) scRNAseq analysis of myoepithelial cell genes *Krt5*, *Krt17, Myc* and *Acta2* expression in human IPF vs donor lungs. Uniform Manifold Approximation and Projection (UMAP) of 10x scRNAseq data on human control and IPF lungs demonstrating high Myc expression in bronchiolized epithelium in IPF. Scale bar: 50μm (A-C), 100μm (D-F).

We next wanted to investigate how Myc levels in BLCs affect bronchiolization. To do this we performed immunostaining for Myc on lungs after H1N1 injury or severe bleomycin injury. We show that after catastrophic H1N1 or severe bleomycin injury some BESC offspring at the periphery or leading edge of the BC-pods feature high Myc levels (Fig.2A–C). Interestingly, these leading edge BLCs unlike trailing cells in the BC pods also express high levels of Sox9 and Acta2 (Fig.2D,E), reminiscent of myoepithelial cells (MECs) in the submucosal gland^38,39^ (SMG). Immunostaining and scRNAseq analysis of human IPF tissue demonstrate that subsets of BCs in honeycomb cysts of IPF lungs also feature high levels of Myc, Sox9 and Acta2 expression (Fig.2F,G).

To investigate if the MEC-like cells at the leading edge of the BC pods are derived from MECs in the SMG, we labeled the latter prior to injury using the *Nkx2*.*1*^*Flpo*^;*Acta2-Frt-STOP-Frt-CreERT2*;*mTmG*^40^ intersectional mouse model in which we can specifically lineage label lung epithelial cells that co-express the lung epithelial cell marker Nkx2.1 and the mesenchymal Acta2 (α-SMA) marker. The *Acta2-Frt-STOP-Frt-CreERT2* knock-in mouse line, possesses a CreERT2 cassette, inserted in the *Acta2* locus, which is preceded by a STOP codon, flanked by Frt sites. As such, when crossed with *Nkx2.1-Flpo* expressing mice, *Acta2-Frt-STOP-Frt-CreERT2* mice permanently express *CreERT2* in *Acta2* and *Nkx2.1-Flpo* co-expressing cells as well as their offspring, as a result of the removal of the STOP codon. This then allows for the lineage labeling of MECs in *Nkx2*.*1*^*Flpo*^;*Acta2-Frt-STOP-Frt-CreERT2*;*mTmG* after tamoxifen treatment.

Using this mouse model we find that SMG MECs do not migrate and give rise to BC pods after H1N1 injury (Fig.S1A,B). However, we can label *de novo* myoepithelial like cells in BC-pods by treating this same intersectional mouse model with tamoxifen after H1N1 injury (Fig.S1C,D), suggesting that BLCs, other than the MECs in the SMG, can acquire MEC-like characteristics upon catastrophic H1N1 or bleomycin injury. We were able to confirm these findings using a different intersectional mouse model *Trp63*^*DreERT2*^*;Acta2*^*CreERT2*^*;RLTG* in which we can specifically target MEC-like cells that co-express the basal cell transcription factor *Trp63* and the mesenchymal *Acta2* (α-SMA) marker. Tamoxifen exposure of these mice results in Dre-mediated excision of a polyA signal (STOP) from the RLTG dual recombinase reporter allele (Dre/Cre recombinase reporter) within *Trp63* expressing intralobular basal cells, with subsequent Cre-mediated excision of *tdT-STOP* within *Acta2*-expressing MECs. Outcomes of these recombination events include tracing of *Trp63*^*+*^-intralobular basal cells by expression of *tdT*, and *Trp63*^*+*^*/Acta2*^*+*^ MECs by expression of *eGFP* (Fig.S1E).

Finally, to investigate whether all cells in BC pods may be derived from these MEC like cells that lead the invasion we performed H1N1 injury on *Sox9*^*CreERT2*^*;mTmG* mice and treated them with tamoxifen chow following injury and found that all cells in the BC pods were lineage labeled (Fig.S1F), indicating they all either induced *Sox9* expression at some point during the invasion of BC pods or are all derived from the MEC-like stem cells at the leading edge of the invasion. This is interesting as it suggests that the process of bronchiolization is reminiscent of the process that drives submucosal gland development, suggesting that BC pods may be considered as *de novo* submucosal glands^41,42^.

### Myc drives bronchiolization and fibrosis through the generation of myoepithelial like cells.

**Figure 3.**
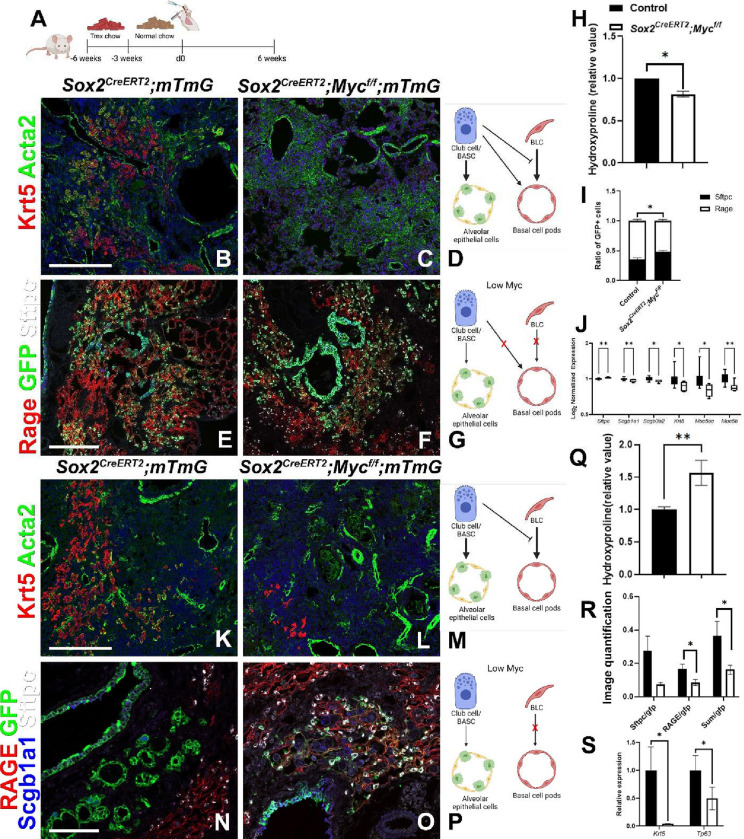
Myc drives bronchiolization through the generation of myoepithelial like cells. (A) Mice were placed on tamoxifen chow for 3 weeks to inactivate Myc and permanently lineage label bronchial epithelial cells and their offspring. Following a 3 week washout period, mice were injured with intratracheal administration of bleomycin (B-J) or intranasal administration of H1N1 (K-S). At 6 weeks post injury, left lung lobes were inflation fixed, embedded in paraffin, and sectioned. (B, C, K, L) Immunostaining for Krt5 (basal and basal-like cells) and Acta2 (smooth muscle actin; myofibroblasts) on bleomycin (B, C) and influenza (K, L) injured *Sox2*^*CreERT2*^*;mTmG* and *Sox2*^*CreERT2*^*;Myc*^*f/f*^*;mTmG*. (E, F, N, O) Immunostaining for Rage (AT1 cells), GFP (lineage label), and Sftpc (AT2 cells) (E, F) and Scgb1a1 (Club cells/BASCs) (N, O) on *Sox2*^*CreERT2*^*;mTmG* and *Sox2*^*CreERT2*^*;Myc*^*f/f*^*;mTmG*. (D, M) Model demonstrating that Myc sufficient Club cells inhibit basal cells and give rise to alveolar epithelial cells after bleomycin (D) and influenza (M) injury. (G, P) Model demonstrating that Myc deficient bronchial epithelial cells fail to give rise to basal cell pods after bleomycin (G) and influenza (P) injury. (H) Hydroxyproline analysis for soluble collagen on bleomycin injured *Cre-* controls (N=26) and *Sox2*^*CreERT2*^*;Myc*^*f/f*^ (n=19). (I) Lineage tracing analysis on bleomycin injured lungs from immunostaining in D (N=3), E (N=5). Area of GFP+/Sftpc+ and GFP+/Rage+ were determined to analyze alveolar regeneration using Aivia artificial intelligence software. Graph depicts the ratio of Sftpc+ and Rage+ of total GFP+ alveolar epithelial cells. (J) Nanostring nCounter analysis on RNA from bleomycin injured *Cre-* controls (N=17) and *Sox2*^*CreERT2*^*;Myc*^*f/f*^*;mTmG* (N=6) lungs for AT2 cell (*Sftpc*) and BLCs (*Scgb1a1, Scgb3a2, Krt5, Muc5ac, Muc5b*) genes. Data are Log2 normalized and graphed as a box and whiskers plot. (Q) Hydroxyproline analysis for soluble collagen on influenza injured *Cre-* controls (N=8) and *Sox2*^*CreERT2*^*;Myc*^*f/f*^ (n=5). (R) Lineage tracing analysis on influenza injured lungs from immunostaining in K (N=5), L (N=5). Area of GFP, Sftpc, and Rage were determined using Aivia artificial intelligence software. Graph depicts the ratio of total area of Sftpc of the total GFP area, ratio of total Rage area of the total GFP area, and the ratio of the sum of Sftpc and Rage area of the total GFP area. (S) qPCR analysis on RNA from influenza injured *Cre-* controls (N=12) and *Sox2*^*CreERT2*^*;Myc*^*f/f*^*;mTmG* (N=7) lungs for BLC genes (*Krt5* and *Tp63*). Scale bars: 100μm. Student’s T test was used to determine significance. *p<0.05, **p<0.01.

To investigate if Myc levels in BESCs affect stem cell competition after severe bleomycin or H1N1 injury we generated *Sox2*^*CreERT2*^*;Myc*^*f/f*^*;mTmG* mice in which we can inactivate *Myc* in all BESCs, in order to level fitness levels, while simultaneously lineage labeling them. When we perform bleomycin or H1N1 injury on *Sox2*^*CreERT2*^*;Myc*^*f/f*^*;mTmG* mice, in which we inactivated *Myc* in BESCs prior to injury (Fig.3A), BESCs fail to acquire MEC-status and fail to bronchiolize the lung parenchyma, as demonstrated by reduced expression of bronchial epithelial markers *Muc5b, Muc5ac* and *Krt5* by Nanostring nCounter RNA analysis (Fig.3B–D,K–M,J,S) and 10x Visium spatial transcriptomics (Fig.S2). Subsets of BESCs in *Sox2*^*CreERT2*^*;Myc*^*f/f*^*;mTmG* mice, presumably Club cells/BASCs, were still able to give rise to alveolar epithelium after severe bleomycin injury but this regeneration was skewed towards the AT2 vs AT1 lineage (Fig.3E–G,N–P,I,J,R) compared to control *Sox2*^*CreERT2*^*;mTmG* mice.

Interestingly, in the bleomycin model, *Sox2*^*CreERT2*^*;Myc*^*f/f*^*;mTmG* lungs feature reduced pulmonary fibrosis based on hydroxyproline content (Fig.3H), suggesting that leveling the fitness of the different BESCs stem cell populations can prevent BASCs from becoming outcompeted/eliminated allowing for regeneration rather than remodeling of the lung parenchyma. However, in the H1N1 injury model in which most BASCs and AT2 stem cells are destroyed we find that that the inability of BLCs to robustly participate in the regenerative response, even though maladaptive, is detrimental to survival (data not shown) and results in increased pulmonary fibrosis (Fig.3Q) compared to control *Cre* negative littermate controls. This suggest that though maladaptive, in certain cases of acute lung injury when proper stem cells have been destroyed this maladaptive repair process is still important to maintain barrier function and survival of the organism.

### Inactivation of Myc in basal cell pods post H1N1 injury promotes their differentiation into AT1 cells.

**Figure 4.**
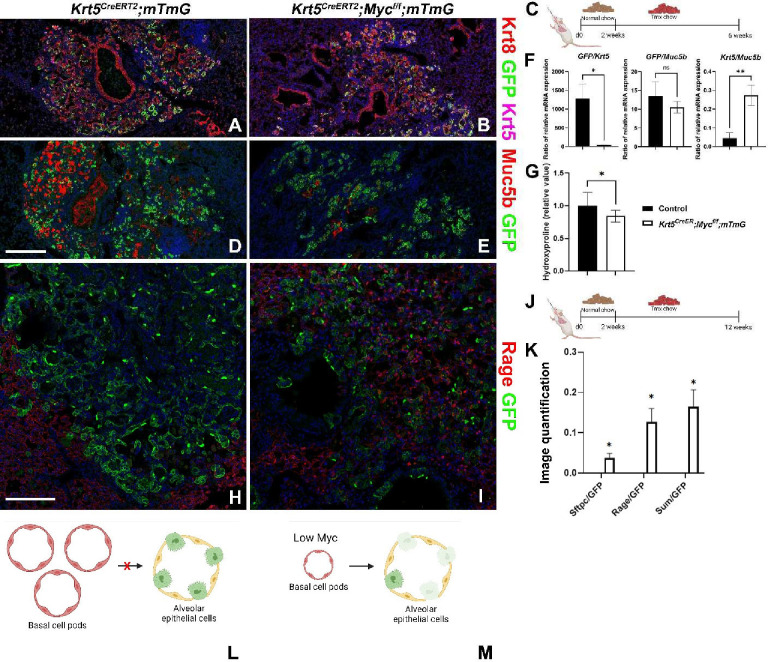
Myc drives basal cell pod expansion after H1N1 injury. (C, J) *Krt5*^*CreERT2*^*;mTmG* and *Krt5*^*CreERT2*^*;Myc*^*f/f*^*;mTmG* were intranasally administered H1N1 at 8 weeks of age. At 2 weeks after injury, mice were placed on tamoxifen chow to inactivate *Myc* and permanently label all BLCs and their offspring with GFP. At 6 (A-G) or 12 (H-M) weeks post injury, left lung lobes were inflation fixed, embedded in paraffin, and sectioned. (A, B) Coimmunostaining for Keratin 8 (Krt8; basal-like cells and transitional cells), GFP (lineage label), and Keratin5 (Krt5; basal and basal-like cells), (D, E) and coimmunostaining for Muc5b (mucus producing secretory cells) and GFP (lineage label) on *Krt5*^*CreERT2*^*;mTmG* (A, D) and *Krt5*^*CreERT2*^*;Myc*^*f/f*^*;mTmG* (B, E). (F) qPCR analysis for Gfp, *Krt5,* and *Muc5b Cre-* control (n=5) and *Krt5*^*CreERT2*^*;Myc*^*f/f*^ (n=5). Values are graphed as ratios. (G) Hydroxyproline analysis for soluble collagen on *Cre-* control (n=10) and *Krt5*^*CreERT2*^*;Myc*^*f/f*^ (n=16). (H-I) Coimmunostaining for Rage (AT1 cells) and GFP (lineage label) on *Krt5*^*CreERT2*^*;mTmG* and *Krt5*^*CreERT2*^*;Myc*^*f/f*^*;mTmG* lungs. (K) Lineage tracing analysis on influenza injured lungs from immunostaining in H and I (N=2). Area of GFP, Sftpc, and Rage were determined using Aivia artificial intelligence software to trace BLC differentiation after H1N1. Graph depicts the ratio of total area of Sftpc of the total GFP area, ratio of total Rage area of the total GFP area, and the ratio of the sum of Sftpc and Rage area of the total GFP area. (L) Model depicting that Myc sufficient basal cells maintain their basal cell fate at 12 weeks after influenza injury. (M) Model depicting that Myc deficient basal cells pods are smaller than Myc sufficient basal cell pods and are capable of differentiating into Rage+ AT1 cells at 12 weeks after influenza injury. Scale bars: 250μm (A, B, D, E) and 125μm (H-I). Student’s T test was used to determine significance. * p<0.05, **p<0.01.

So far, our findings suggest that Myc levels in lung stem cells determine their fitness levels and that cells with the lowest Myc levels differentiate into AT1 cells. Since BC-pods are known to persist in the lung long after H1N1 infection, we wondered whether *Myc* is required for their maintenance and/or expansion post H1N1 injury. To investigate this we infected *Krt5*^*CreERT2*^*;mTmG* and *Krt5*^*CreERT2*^*;Myc*^*f/f*^*;mTmG* mice with H1N1 influenza, and lineage labeled their BLCs with or without simultaneous inactivation of *Myc,* starting at 2 weeks after injury (Fig.4C). Interestingly, we find that upon inactivation of *Myc* in BC pods in *Krt5*^*CreERT2*^*;Myc*^*f/f*^*;mTmG* mice,BC-pods are reduced in size, as indicated by less GFP RNA per Krt5 transcript, compared to *Krt5*^*CreERT2*^*;mTmG* mice (Fig.4A–F). In addition, we find that compared to H1N1 injured *Krt5*^*CreERT2*^*;mTmG* mice, fibrosis is reduced in *Krt5*^*CreERT2*^*;Myc*^*f/f*^*;mTmG* mice, in which we inactivated *Myc* in BLCs and MEC-like cells after injury (Fig.4G). More strikingly we find that inactivation of *Myc* in BC pods post H1N1 injury affects BC stem cell maintenance over time and allows for their differentiation towards the AT1 lineage by 12 weeks after H1N1 injury (Fig.4H–M).

### Overexpression of *Myc* endows subsets of BESCs with a super competitor myoepithelial like status which can outcompete AT2 stem cells.

We next wondered what would happen if we boosted the fitness of Club cells by overexpressing *Myc* in Club cells after a less severe bleomycin injury. Overexpression of a dominant active version of the Hippo transcriptional effector *Yap*^*S112A*^ in Club cells has been shown to direct their differentiation along the BCL lineages^43^, and Myc and Yap have both been shown to be important for cell competition^26,28,33,44,45^.

**Figure 5.**
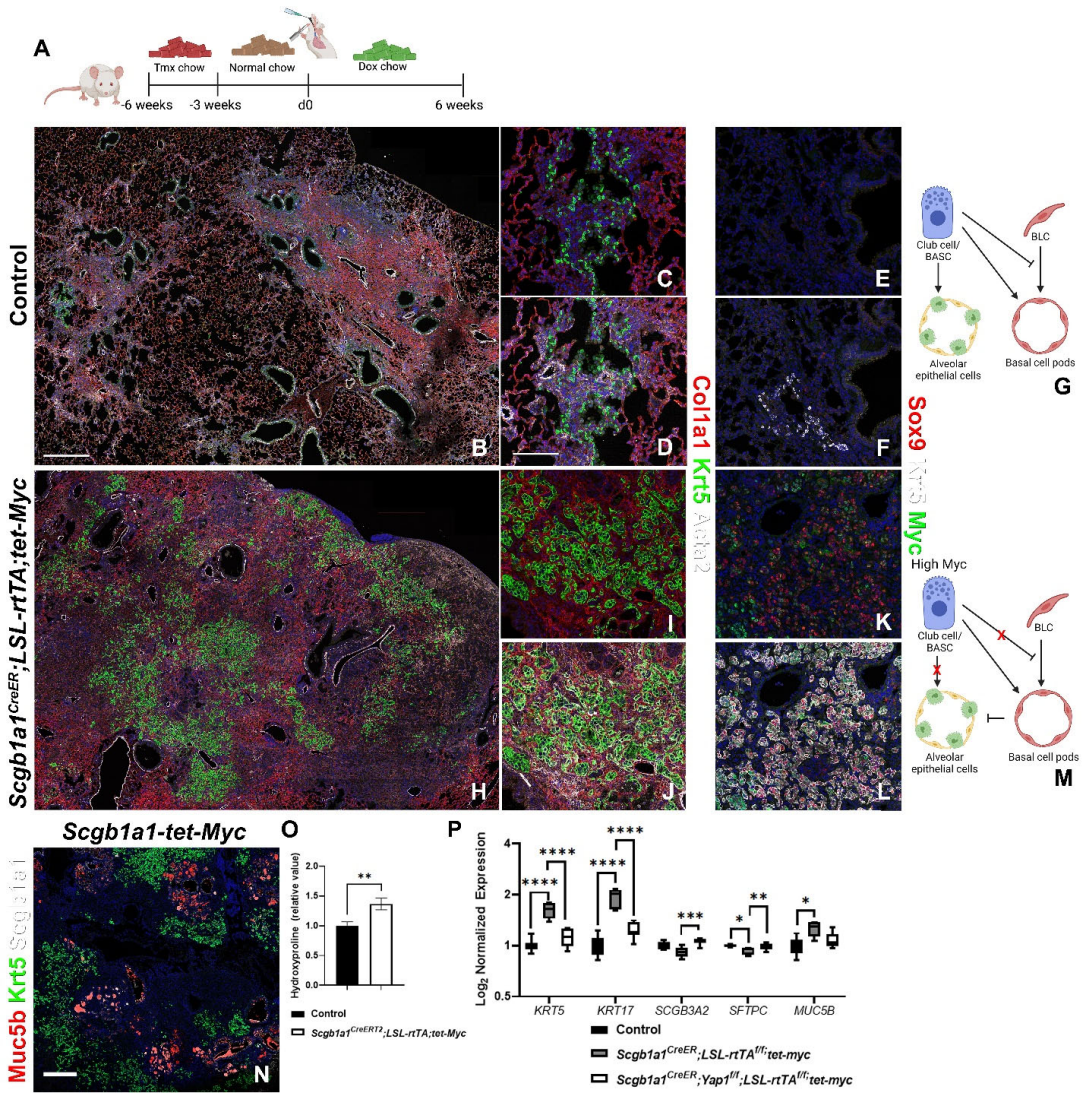
*Myc* overexpression in bronchial epithelial stem cells promotes SCMC status. (A) Mice were placed on tamoxifen chow for 3 weeks and following a 3 week washout period, mice were injured with intratracheal administration of bleomycin and placed on doxycycline containing chow to induce *Myc* overexpression. At 6 weeks post injury, left lung lobes were inflation fixed, embedded in paraffin, and sectioned. (B-D, H-J) Coimmunostaining for markers associated with fibrosis and bronchiolization (Col1a1, Krt5, and Acta2) on control (B-D) and *Scgb1a1*^*CreERT2*^*;LSL-rtTA;Tet-Myc* (H-J). (E, F, K, L) Coimmunostaining for myoepithelial cell-like markers Sox9, Krt5, and Myc on control (E-F) and *Scgb1a1*^*CreERT2*^*;LSL-rtTA;Tet-Myc* (K-L). (G) Model demonstrating that Myc sufficient Club cells inhibit basal cells and give rise to alveolar epithelial cells after bleomycin injury. (M) Model demonstrating that Myc overexpressing Club cells dedifferentiate into basal cells and promote their invasion into the alveolus. (N) Immunostaining for Muc5b (mucus producing secretory cells), Krt5 (BLCs), and Scgb1a1 (Club cells/BASCs) on *Scgb1a1*^*CreERT2*^*;LSL-rtTA;Tet-Myc*. (O) Hydroxyproline analysis for soluble collagen on *Cre-* control and *Scgb1a1*^*CreERT2*^*;LSL-rtTA;Tet-Myc* (n=47) normalized to control. (P) Log_2_ normalized values for RNA expression for BLC (*Krt5, Krt17, Scgb3a2, Muc5b*) and AT2 cell (*Sftpc*) genes NanoString analysis on control (n=9), *Scgb1a1*^*CreERT2*^*;LSL-rtTA;Tet-Myc* (n=5), and *Scgb1a1*^*CreER*^*;Yap*^*f/f*^*;LSL-rtTA;Tet-Myc* (n=8). Values are normalized to control and graphed as a box and whiskers plot. Scale bars: 500μm (B,G,L) and 100 μm (C-F,H-K). Student’s T test was used to determine significance. * p<0.05, **p<0.01, ***p<0.001, ****p<0.0001.

Interestingly, when we overexpress *Myc* in Club cells/BASCs after bleomycin injury (Fig.5A), Club cells/BASCs massively acquire a super-competitor myoepithelial cell (SCMC) like status, coexpressing Krt5, Acta2, Sox9 and Myc (Fig.5B–M), resulting in the hyper-invasion and apparent destruction of the lung parenchyma including AT2 cells and its replacement with bronchial epithelial cells demonstrated by increased expression of bronchial epithelial markers *Krt5, Trp63, Krt17, Muc5ac, and Muc5b,* increased pulmonary fibrosis, based on hydroxyproline content and reduced expression of alveolar epithelial markers (*Ager* and *Sftpc*) by Nanostring nCounter RNA analysis (Fig.5B–P) and 10x Visium spatial transcriptomics (Fig.S3). This suggests that the cell competition program may converge onto a SCMC plastic like state that can be acquired by different BESC populations.

### Lung epithelial stem cell fitness levels are determined by their Myc levels, which are tuned by the Hippo pathway.

It is well known that Hippo pathway plays an important role in cell competition, and Yap and Myc are thought to work together in this process^26,28,33,44,45^. It is also well known that *Myc* is the quintessential target gene of the canonical Wnt signaling pathway^46^ and that the Hippo pathway controls β-catenin stabilization and nuclear localization^47^. However, how the Hippo pathway affects Myc levels seems to be context dependent.

**Figure 6.**
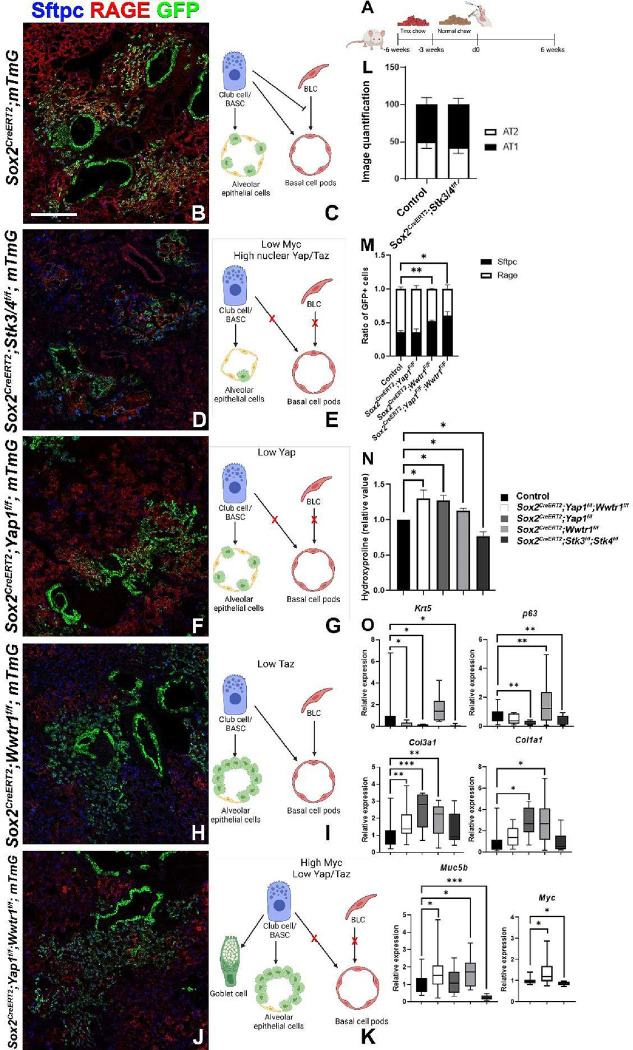
Cytoplasmic Yap/Taz in airway epithelial cells inhibits bronchiolization and pulmonary fibrosis. (A) Mice were placed on tamoxifen chow for 3 weeks to inactivate *Yap1* and/or *Wwtr1* or *Stk3/4* and permanently label all Sox2+ cells and their offspring with GFP. Following a 3 week washout period, mice were injured with intratracheal administration of bleomycin. At 6 weeks post injury, left lung lobes were inflation fixed, embedded in paraffin, and sectioned. (B, D, F, H, J) Coimmunostaining for Rage (AT1 cells), GFP (lineage label), and Sftpc (AT2 cells) on *Sox2*^*CreERT2*^*;mTmG* (B), *Sox2*^*CreERT2*^*;Stk3*^*f/f*^*;Stk4*^*f/f*^*;mTmG* (D), *Sox2*^*CreERT2*^*;Yap1*^*f/f*^*;mTmG* (F), *Sox2*^*CreERT2*^*;Wwtr1*^*f/f*^*;mTmG* (H), and *Sox2*^*CreERT2*^*;Yap1*^*f/f*^*;Wwtr1*^*f/f*^*;mTmG* (J) lungs. (C) Model demonstrating that Myc sufficient/Hippo active Club cells inhibit basal cells and give rise to alveolar epithelial cells after bleomycin injury. (E) Model demonstrating that Mst1/2 deficient bronchial epithelial cells have increased *Myc* expression and nuclear Yap/Taz, inhibit basal cell pods and give rise to alveolar epithelial cells (primarily AT1 cells) after bleomycin injury. (G) Model demonstrating that Yap deficient bronchial epithelial cells fail to give rise to basal cells and instead give rise to alveolar epithelial cells after bleomycin injury. (I) Model demonstrating that Taz deficient bronchial epithelial cells give rise to basal cell pods and give rise AT2 epithelial cells but not AT1 cells after bleomycin injury. (K) Model demonstrating that Yap/Taz deficient bronchial epithelial cells have increased Myc expression, differentiate into goblet cells, fail to give rise to basal cells and give rise AT2 epithelial cells at the expense of AT1 cells after bleomycin injury. (L) Lineage tracing analysis on bleomycin injured lungs from immunostaining in B (n=3) and C (n=4). Area of GFP, Sftpc, and Rage were determined using Zeiss Zen Intellesis artificial intelligence software to trace. The total area of alveolar regeneration was determined by analyzing the total area GFP labeled AT2 and AT1 cells were determined. Graph depicts the ratio of total area of Sftpc of the total area of alveolar regeneration and ratio of total Rage area of the total area of alveolar regeneration as a total of alveolar regeneration. (M) Lineage tracing analysis on bleomycin injured lungs from immunostaining in B (n=3), D (n=5), E (n=4), and F (n=4). Area of GFP+/Sftpc+ and GFP+/Rage+ were determined to analyze alveolar regeneration using Aivia artificial intelligence software. Graph depicts the ratio of Sftpc+ and Rage+ of total GFP+ alveolar epithelial cells. N) Hydroxyproline analysis for soluble collagen on *Cre-* control, *Sox2*^*CreERT2*^*;Yap1*^*f/f*^*;Wwtr1*^*f/f*^ (n=19), *Sox2*^*CreERT2*^*;Yap1*^*f/f*^ (n=25), *Sox2*^*CreERT2*^*;Wwtr1*^*f/f*^ (n=9), and *Sox2*^*CreERT2*^*;Stk3*^*f/f*^*;Stk4*^*f/f*^ (n=18). (O) qPCR analysis for bronchiolization and fibrosis genes (*Krt5, p63, Muc5b, Col1a1,* and *Col3a1*) on *Cre-* control (n=22), *Sox2*^*CreERT2*^*;Yap1*^*f/f*^*;Wwtr1*^*f/f*^ (n=8), *Sox2*^*CreERT2*^*;Yap1*^*f/f*^ (n=5), *Sox2*^*CreERT2*^*;Wwtr1*^*f/f*^ (n=10), and *Sox2*^*CreERT2*^*;Stk3*^*f/f*^*;Stk4*^*f/f*^ (n=10) and *Myc* on control (n=23), *Sox2*^*CreERT2*^*;Yap1*^*f/f*^*;Wwtr1*^*f/f*^ (n=14) and *Sox2*^*CreERT2*^*;Stk3*^*f/f*^*;Stk4*^*f/f*^ (n=10). Values are represented as 2^−ΔΔCt^ normalized to Control. Values are normalized to control. Scale bar: 200μm. Student’s T test was used to determine significance. * p<0.05, **p<0.01, ***p<0.001.

Interestingly, the Hippo pathway is active in AT2^48^ and Club cells (Fig.S5A–E) resulting in the degradation and cytoplasmic retention of Yap and Taz the nuclear effectors of the pathway. To investigate how increased Yap/Taz levels may affect *Myc* expression in BESCs or AT2 cells we inactivated *Merlin* (encoded by *Nf2*), one of the most upstream activators of the Hippo pathway or inactivated the Hippo kinases *Mst1/2* (encoded by *Stk4/3*) in BESCs, and found that this resulted in decreased *Myc* expression, decreased bronchiolization, increased AT1 cell regeneration and reduced pulmonary fibrosis based on hydroxyproline content, upon severe bleomycin injury (Fig.6A–E,L,N,O, Fig.S4).

Interestingly, inactivation of the Hippo pathway in Club cells or AT2 stem cells in the absence of injury results in their spontaneous differentiation into AT1 cells, consistent with previous reports^49,50^ (Fig.S5). Vice versa, inactivation of the Hippo nuclear effector genes *Yap1* and *Wwtr1* in BESCs, using *Sox2*^*CreERT2*^*;Yap1*^*f/f*^*;Wwtr1*^*f/f*^*;mTmG* mice resulted in increased *My*c expression, decreased alveolar epithelial regeneration and increased pulmonary fibrosis based on hydroxyproline content upon severe bleomycin injury (Fig.6J,K,N,O). Even though *Sox2*^*CreERT2*^*;Yap1*^*f/f*^*;Wwtr1*^*f/f*^*;mTmG* mice featured increased *Myc* levels, their lack of Yap prevented them from BLC mediated bronchiolization (Fig.6J,K,O). Instead, *Sox2*^*CreERT2*^*;Yap1*^*f/f*^*;Wwtr1*^*f/f*^*;mTmG* mice featured increased goblet cell differentiation based on *Muc5b* expression (Fig.6O), something we also observed in the absence of injury (Fig.S5H,I,L), and which is consistent with previous reports^51^ demonstrating the spontaneous differentiation of BESCs into goblet cells upon simultaneous inactivation of *Yap1* and *Wwtr1*.

Together these findings suggest that the Hippo pathway controls Myc levels and therefore stem cell competitiveness by controlling Yap/Taz levels. This is consistent with a role for Yap/Taz in promoting β-catenin degradation in the cytoplasm^47^. Interestingly, overexpression of a dominant active *β-catenin(ex3)* in the airway epithelium also results in excessive goblet cell differentiation^52^.

### Yap-Myc-p63 promote bronchiolization whereas Taz promotes AT1 differentiation.

The fact that inactivation of *Nf2* or *Stk4/3* in Club or AT2 cells results in their spontaneous differentiation into AT1 cells is intriguing since we and others have previously demonstrated that Yap is required for tracheal BC maintenance and overexpression of a dominant active version of the Hippo transcriptional effector *Yap*^*S112A*^ in Club cells is able to drive their differentiation towards a BLC lineage in cooperation with p63. Together all these findings suggest a role for Yap-Myc-Trp63 in the acquisition of SCMC state whereas a lack of Yap-Myc-Trp63 promotes AT1 cell differentiation.

**Figure 7.**
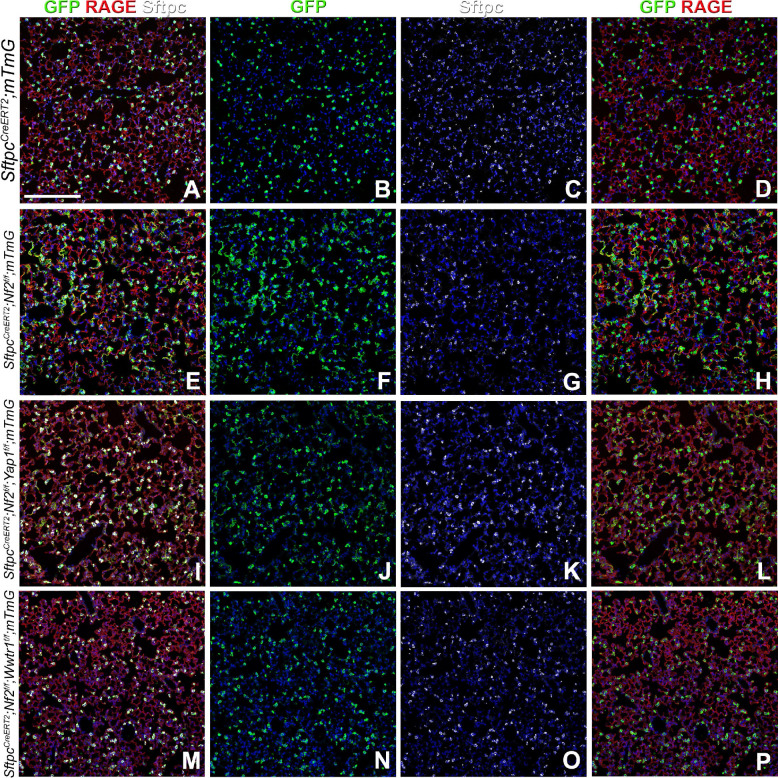
Taz is required for AT1 cell differentiation. *Sftpc*^*CreERT2*^*;mTmG, Sftpc*^*CreERT2*^*;Nf2*^*f/f*^*;mTmG, Sftpc*^*CreERT2*^*;Nf2*^*f/f*^*;Yap1*^*f/f*^*;mTmG,* and *Sftpc*^*CreERT2*^*;Nf2*^*f/f*^*;Wwtr1*^*f/f*^*;mTmG* were placed on tamoxifen containing chow at 8 weeks of age for 3 weeks to inactivate *Nf2* and/or *Yap1* and/or *Wwtr1* and permanently lineage label AT2 stem cells and their offspring. At 9 weeks after being placed on normal chow left lung lobes were inflation fixed, embedded in paraffin, and sectioned. Coimmunostaining for GFP (lineage label), Rage (AT1 cells), and Sftpc (AT2 cells). Scale bar: 200μm.

Interestingly, though we have long favored a role for Taz and not Yap in AT1 cell differentiation and maintenance, some reports seem to suggest a role for Yap in AT1 cell differentiation. To definitively answer this question we generated *Sftpc*^*CreERT2*^*;Nf2*^*f/f*^*;Wwtr1*^*f/f*^*;mTmG* and *Sftpc*^*CreERT2*^*;Nf2*^*f/f*^*;Yap1*^*f/f*^*;mTmG* mice to investigate which nuclear effector of the Hippo pathway is required for the spontaneous differentiation of AT2 cells into AT1 cells upon *Nf2* inactivation. We now demonstrate that the simultaneous inactivation of *Nf2* and *Yap* in AT2 cells does not affect their spontaneous differentiation into AT1 cells (Fig.7A–L), whereas the simultaneous inactivation of *Nf2* and *Taz* in AT2 cells completely blocks this process (Fig.7A–P).

If Taz promotes AT1 cell differentiation and Yap promotes BLC differentiation, inactivation of *Taz* in bronchial epithelium using *Sox2*^*CreERT2*^*;Wwtr1;mTmG* mice should mainly affect AT1 cell differentiation but not bronchiolization, whereas inactivation of *Yap1* in airways using *Sox2*^*CreERT2*^*;Yap1*^*f/f*^*;mTmG* mice should only affect bronchiolization. Indeed *Sox2*^*CreERT2*^*;Yap1*^*f/f*^*;mTmG* mice fail to generate BLCs and to bronchiolize the lung parenchyma upon H1N1 injury or severe bleomycin injury (Fig.6F,G,O & Fig.S6A–I). However, *Sox2*^*CreERT2*^*;Yap1;mTmG* mice also featured increased mortality and pulmonary fibrosis upon H1N1 injury (Fig.S6A–I), as measured by hydroxyproline content similar to what we observed in *Sox2*^*CreERT2*^*;Myc;mTmG* mice. Suggesting again that the maladaptive repair of the lung parenchyma through bronchiolization is necessary for organism survival upon acute lung injury by H1N1.

Since overexpression of dominant active *Yap*^*S112A*^ in BESCs is sufficient to drive Club cell to BLC differentiation, we wondered if overexpression of dominant active *Yap*^*S112A*^ alone in BESCs and their offspring after bleomycin injury, using *Sox2*^*CreERT2*^*;LSL-rtTA;Tet-Yap1*^*S112A*^ mice is sufficient to promote bronchiolization. Interestingly, while overexpression of a dominant active *Yap*^*S112A*^ in BESCs is sufficient to drive BLC differentiation and prevent alveolar epithelial differentiation after bleomycin injury (Fig.S6H–N), overexpression of dominant active *Yap*^*S112A*^ did not induce *Myc* expression, and BESCs failed to acquire SCMC status and as such did not amplify nor invade the lung parenchyma nor destroy the remaining alveolar epithelium (Fig.S6H–N).

This is interesting as we have just demonstrated that overexpression of *Myc* in Club cells after bleomycin injury causes them to acquire a super-competitor myoepithelial cell (SCMC) like status, coexpressing Krt5, Acta2, Sox9 and Myc (Fig.5). Therefore, to specifically investigate the requirement for Yap and Myc in the acquisition of MEC-status we generated *Scgb1a1*^*CreER*^*;Yap*^*f/f*^*;LSL-rtTA;Tet-Myc* mice in which we could simultaneously inactivate *Yap1* and overexpress *Myc* in Club cells after bleomycin injury and found that BCs and MEC-like cells development was impaired, indicating that that both Myc and Yap are required for obtaining SCMC status (Fig.5P).

Finally, to investigate if AT2 stem cells have the capacity to acquire a SCMC state and to bronchiolize the lung parenchyma we generated *Sftpc*^*CreERT2*^*;LSL-rtTA;Tet-Myc*, *Sftpc*^*CreERT2*^*;LSL-rtTA;Tet-Yap1*^*S112A*^ and *Sftpc*^*CreERT2*^*;LSL-rtTA;Tet-Myc;Tet-Yap1*^*S112A*^ mice in which we could respectively overexpress *Myc*, a dominant active *Yap*^*S112A*^ or both together in AT2 stem cells and their progeny after bleomycin injury. We found that simultaneous overexpression of both *Myc* and *Yap1*^*S112A*^ in AT2 cells allowed them to adopt SCMC state and to bronchiolize the lung parenchyma (Fig.S7A–D). However, overexpression of *Myc* or *Yap1*^*S112A*^ alone was not sufficient (Fig.S7E–L).

## Discussion

In this manuscript, we set out to investigate and clarify several unresolved issues on lung Hippo signaling, ARDS and pulmonary fibrosis. We find that different lung stem cell populations compete with one another to regenerate the lung and that this stem cell competition follows the classical cell competition models originally identified in *Drosophila*^53,54^. Our findings have wide ranging implications for the field of lung regeneration and fibrosis in particular. Our model in which bronchiolization of the lung parenchyma is reminiscent of the development of submucosal glands, has wide ranging implications for the early diagnosis of pulmonary fibrosis as well development of new treatments for this devastating disease. Especially, our findings about the distinct roles for Yap and Taz in bronchiolization vs alveolar epithelial regeneration will allow for the development of targeted therapies.

It is thought that Yap and Myc coordinately regulate genes required for cell proliferation, where activation of Myc leads to extensive association with its genomic targets, most of which are prebound by TEAD^55^. At these loci, recruitment of Yap is thought to be Myc-dependent and required for full transcriptional activation. This cooperation between Yap and Myc is thought to be critical for cell cycle entry, organ growth, and tumorigenesis^55^. Cells could become super competitors through intrinsic (e.g., somatic mutations) or extrinsic mechanism^56^. At the molecular level, future studies will need to explore the impact of genetic perturbations on the ability of winner cells to contribute to cellular populations, both *in vitro* and *in vivo*. However, changes to gene expression shown to drive cell competition need not involve genetic engineering or mutations to the DNA itself. Cells may receive signals from their microenvironment, including cell-cell interactions, that converge on the cellular processor and drive cell competition behavior by affecting gene expression^57–59^. Similarly, epigenetic changes can also drive cell competition-relevant gene expression changes.

Under normal conditions, cell competition will select against the emergence of altered cells with disruptive behavior towards tissue integrity and/or tissue pattern formation. However, upon catastrophic organ injury this molecular machinery involved in the winner/loser interaction could be hijacked to maintain organism survival. In idiopathic pulmonary fibrosis (IPF) and acute respiratory distress syndrome (ARDS), such as experienced after influenza or SARS-CoV-2 infection, normal alveolar tissue is steadily being replaced by bronchial/conducting airway epithelial cells which cannot participate in gas exchange. Stem cells in the conducting airway are sometimes considered a “reserve stem cell” population that only participates in alveolar epithelial repair after catastrophic injury to the lung parenchyma. As such these “reserve stem” cells only win the fitness battle upon loss or destruction of alveolar type 2 (AT2) stem cells, considered the “dominant” stem cell population in the alveolar compartment^24^. We recently also reported increased bronchiolization in mice in which the fitness of AT2 cells was compromised^48^. Therefore, it appears that lowering AT2 stem cell fitness can be sufficient to cause conducting airway epithelial stem cells to acquire a competitive advantage and drive bronchiolization.

We demonstrate that active cell competition is a feature of pulmonary fibrosis/ARDS and its underlying mechanisms can be manipulated to help prevent and treat this disease. Lowering the fitness of BESCs can reduce and even reverse pulmonary fibrosis progression. Boosting the fitness/survival of AT2 stem cells or BASCs, could also prevent bronchiolization. Therefore, cell competition can be exploited to maximize the potential of healthy tissue replacement.

## Material and methods

### CONTACT FOR REAGENT AND RESOURCE SHARING

Further information and requests for resources and reagents should be directed to and will be fulfilled by the Lead Contact, Stijn De Langhe: delanghe.stijn@mayo.edu

#### Experimental model and subject details

All mice were bred and maintained in a pathogen-free environment with free access to food and water. Both male and female mice were used for all experiments. *Sox2*^*CreERT2*^ (JAX 017593; RRID:IMSR_JAX:017593), *Krt5*^*CreERT2*60^, *Scgb1a1*^*CreER*^ (JAX 016225 RRID:IMSR_JAX:016225), *Sftpc*^*CreERT261*^, *Trp63*^*DreERT2*^ (Shanghai Model organisms Center NM-KI-190029 RRID:IMSR_NM-KI-190029), *Acta2*^*CreERT262*^, *Sox9*^*CreERT2*^ (JAX 035092 RRID:IMS9R_JAX:035092), *mTmG* (JAX 007676; RRID:IMSR_JAX:007676), Rosa26-tdTomato (JAX 007909 RRID:IMSR_JAX:007909), RLTG (JAX 026931RRID:IMSR_JAX:026931), *Stk3/4*^*f/f*^ (JAX 017635; RRID:IMSR_JAX:017635), *Yap1*^*f/f*63^, *Wwtr1*^*f/f*63^,*Nf2*^*f/f*64^, *Myc*^*f/f*65^, *Rosa26-CAGs-LSL-rtTA3* (LSL-rtTA^f/f^; JAX 029617; RRID:IMSR_JAX:029617), *Tet-Yap1-H2BGFP* (JAX 031279; RRID:IMSR_JAX:031279), *Tet-myc* (JAX 019376; RRID:IMSR_JAX:019376) *Acta2-Frt-STOP-Frt-Cre*^*ERT240*^, *Nkx2.1*^*Flpo*^ (JAX 028577; RRID:IMSR_JAX:028577) mice were previously described.

For bleomycin injury, adult 8- to 12-week-old mice were intratracheally instilled with 50 uL bleomycin (0.8–2 U/kg body weight optimized for each strain, batch of bleomycin, and gender) as described previously^66^. The following reagent was obtained through BEI Resources, NIAID, NIH: Influenza A Virus, A/Puerto Rico/8–9VMC3/1934 (H1N1), NR-29028. Mice were infected via intranasal route with a sublethal dose of H1N1 (100,000–162,500 viral forming units (VFU) optimized for each strain) diluted in 50μL of saline. For tamoxifen induction, *Sftpc*^*CreERT2*^, *Scgb1a1*^*CreER*^ and *Sox2*^*CreERT2*^ mice were placed on tamoxifen containing chow (rodent diet with 400 mg/kg tamoxifen citrate; Harlan Teklad TD.130860) for 3 weeks and *Sftpc*^*CreERT2*^ and *Sox2*^*CreERT2*^ mice received an additional intraperitoneal tamoxifen injection (0.20 mg/g body weight, Millipore Sigma) in the last week of tamoxifen citrate feed. Following a 3 week tamoxifen washout period, mice were either injured with bleomycin or H1N1. Mice containing *LSL-rtTA3* were placed on doxycycline containing chow (rodent diet with 625 mg/kg doxycycline; Harlan Teklad TD.09761) on the day of bleomycin. *Sox9*^*CreERT2*^, *Trp63*^*DreERT2*^*;Acta2*^*CreERT2*^, and *Krt5*^*CreERT2*^ mice were placed on tamoxifen containing chow beginning at 2 weeks following injury. All experiments were approved by the Mayo Clinic Institutional Animal Care and Use Committee.

#### Immunohistochemistry and fluorescence

All staining was done on paraffin sections of formalin-fixed lungs. Immunofluorescent staining was performed with the following primary antibodies: rabbit anti-Merlin (NF2; 1:250; clone A-19; sc-331; RRID:AB_2298548; Santa Cruz Biotechnology), rabbit anti-phosporylated-Mst1(Thr183)/2(Thr180) (1:200; 3681; RRID:AB_330269; Cell Signaling Technologies), rabbit anti-phosphorylated Yap (Ser127) (1:200; 4911; RRID:AB_2218913; Cell Signaling Technologies), goat anti-Scgb1a1 (1:200; clone T-18; sc-9772; RRID:AB_2238819; Santa Cruz Biotechnology Inc.), goat anti-Sox9 (1:500; AF3075; RRID:AB_2194160; R&D Systems), mouse anti-Keratin 17 (1:50; clone Ks17.E3; sc-101461; RRID:AB_2234376; Santa Cruz Biotechnology, Inc.), chicken anti-GFP (1:500; GFP-1020; RRID:AB_10000240; Aves Labs Inc.), rabbit anti-Keratin 5 (1:200; clone EP1601Y; MA5–14473; RRID:AB_10979451; Thermo Fisher Scientific), chicken anti-Keratin 5 (1:500; 905901; RRID:AB_2565054; BioLegend), rabbit anti-SFTPC (1:200; WRAB-9337; RRID:AB_2335890; Seven hills bioreagents), rat anti-RAGE (1:500; Clone 175410; MAB1179; RRID:AB_2289349; R&D Systems), goat anti-RAGE (1:500; AF1145; RRID:AB_354628; R&D Systems), rat anti-keratin 8 (1:100; TROMA-I; RRID:AB_531826; Developmental Studies Hybridoma Bank), Syrian hamster anti-podoplanin (PDPN, T1a; 1:500; 8.1.1; RRID:AB_531893; Developmental Studies Hybridoma Bank), rabbit anti-mucin 5b (Muc5b; 1:250; clone H-300; sc-20119; RRID:AB_2282256; Santa Cruz Biotechnology Inc.), mouse anti-alpha actin (smooth muscle actin (SMA), Acta2; 1:500; Clone 1A4; sc-32251; RRID:AB_262054; Santa Cruz Biotechnology Inc.), rabbit anti-myc (1:200; clone Y69, ab32072; RRID:AB_731658; Abcam), rabbit anti-collagen I (1:500; ab34710; RRID:AB_731684; Abcam), mouse anti–beta-tubulin (1:500; clone 3F3-G2; LMAB-3F3; RRID:AB_451728; Seven Hills Bioreagents), mouse anti-beta-catenin (1:100, 610154, RRID:AB_397555; BD Biosciences), and rabbit anti-p63 (1:500; clone poly6190; 619002; RRID: AB_2207170; BioLegend).

After deparaffinization, slides were rehydrated through a series of decreasing ethanol concentrations, antigen unmasked by either microwaving in citrate-based antigen unmasking solution (Vector Labs, H-3300) or by incubating sections with proteinase K (7.5μg/ml) (Invitrogen, 25530–049) for 7 min at 37°C. Tissue sections were then washed in TBS with 0.1% Tween-20 and blocked with 3% Bovine Serum Albumin (BSA), 0.4% Triton X-100 in TBS for 30 min at room temperature followed by overnight incubation of primary antibodies diluted in 3% BSA, 0.1% Triton X-100 in TBS. The next day, slides were washed in TBS with 0.1% Tween-20 and incubated with secondary antibodies diluted in 3% BSA, 0.1% Triton X-100 in TBS for 3h at room temperature. All fluorescent staining was performed with appropriate secondary antibodies from Jackson Immunoresearch. Slides were mounted using Vectashield (Vector Labs, H-1000).

#### Microscopy and imaging

Tissue was imaged using a micrometer slide calibrated Zeiss LSM800 Laser scanning confocal microscope using ZEN imaging software or Leica Stellaris 5 confocal microscope with LASX imaging software. Lungs were imaged using tiled stitched 20x images covering the entire cross-section of the left or lower right lung lobe from ≥6 different lungs. Representative images were chosen. Images were processed and analyzed using Zen blue (Zeiss), LASX (Leica) and Adobe Photoshop 2024 (Adobe) software.

#### Image quantification

Differentiation of GFP positive cells was determined using artificial intelligence and machine learning image segmentation with Aivia software. The total area of GFP and GFP overlapping with different cell specific antibody stains (Sftpc or RAGE) was determined. Image quantification and analysis was performed in a double blinded fashion. Each quantification was ≥3 different mouse lungs.

#### Quantitative real-time PCR

Total mRNA was extracted from lung accessory lobes stored in RNALater (Invitrogen, AM7021) and using Total RNA Kit I (Omega Biotek, R6834–02) according to the manufacturer’s instructions. RNA concentration was determined by spectrophotometry. cDNA was generated using Maxima^™^ First Strand cDNA Synthesis (Fisher Scientific, FERK1642) according to the manufacturer’s instructions. Gene expression was analyzed by quantitative RT-PCR using Taqman Gene Expression Assays (Applied Biosystems, 4369016) directed against the mouse targets *β-glucuronidase* (Mm00446953_m1), *Krt5* (Mm01305291_g1), *Trp63* (Mm00495788_m1), *Muc5b* (Mm00466391_m1), *Col1a1* (Mm00801666_g1), *Col3a1* (Mm01254476_m1), *Myc* (Mm00487803_m1). Quantitative real-time PCR was performed using a StepOne Plus system (Applied Biosystems). Data were presented as 2^−ΔΔCt^ with *β-glucuronidase* as the internal sample control normalized to control group. Each experiment was repeated with samples obtained from ≥3 different lung preparations.

#### Nanostring

RNA was isolated from lung accessory lobes as described above. 100ng of RNA was hybridized with a custom RNA probe panel designed by NanoString (NanoString Technologies) for 16 hours according to manufacturer’s instructions. The RNA-probe hybridization was loaded on a NanoString cartridge and processed in a NanoString nCounter. Data was analyzed with Rosalind.bio (Rosalind, Inc) and Log2 Fold Changes were calculated and graphed. Each experiment was repeated with samples obtained from ≥3 different lung preparations.

##### Single Cell RNA sequencing of Human IPF tissue.

Epithelial cells from donor distal samples and IPF fibrotic samples were subset from GSE132914 for this analysis^67^. Standard data integration workflow from Seurat V3 package was applied to integrate and combine data sets for unsupervised clustering. The batch correction was processed with PCA (Principal Component Analysis) using the 5000 most variable genes, and the first 30 independent components were used for downstream unbiased clustering with a resolution of 0.4. The UMAP (Uniform Manifold Approximation and Projection) method was used for visualization of unsupervised clustering and basal cell subset with the first 30 independent PCA components. The cell type of each cluster is determined by known markers of individual cell types. Gene expression levels were shown using FeaturePlot function from Seurat Package.

#### Spatial transcriptomics

RNA was isolated from formalin fixed paraffin embedded (FFPE) tissue sections using E.Z.N.A FFPE RNA Kit (Omega Bio-Tek). The RNA integrity in FFPE blocks was determined on an Agilent TapeStation. 5μm FFPE lung sections that had a DV200% above 50 were placed within the frames of the capture areas on the active surface of the Visium spatial slide (10x Genomics) and processed according to manufacturer’s instructions. Tissues were stained with podoplanin (PDPN, T1a) and GFP and imaged with fluorescent secondary antibodies. Final library preparations and sequencing were completed by the Mayo Genomics Research Core according to manufacturer’s instructions on an Illumina NextSeq. Count matrices were generated using the ‘spaceranger count’ function in Space Ranger 1.0.0. The resulting data were processed in Scanpy, Squidpy and Decoupler. The Decoupler DoRothEA wrapper was used to predict transcription factor activity. DoRothEA is a comprehensive resource containing a curated collection of transcription factors (TFs) and their target genes.

#### Hydroxyproline

The right lobes were flash frozen in dry ice at the time of harvest and stored at −80 °C. For acid hydrolysis, the lobes were baked in a 70°C oven without lids for 2 days until completely dry. The weights of dry lobes were measured and 500μl of 6N HCl were added to each sample. The lungs were then hydrolyzed in an 85°C oven for 2 days with occasional vortexing. The hydrolysates were cooled at room temperature and centrifuged at maximum speed for 10 minutes. The supernatants then were transferred to fresh 1.5 mL tubes and centrifuged at maximum speed for 10 minutes. Each sample or standard was diluted with citrate-acetate buffer (5% citric acid, 1.2% glacial acetic acid, 7.24% sodium acetate, and 3.4% sodium hydroxide) in a 96-well plate. Chloramine-T solution (1.4% chloramine-T, 10% N-propanol, and 80% citrate-acetate buffer) was added, and the mixture was incubated for 20 minutes at room temperature. Then, Ehrlich’s solution (1.27M p-dimethylaminobenzaldehyde, 70% N-propanol, 20% perchloric acid) was added to each sample and the samples were incubated at 65°C for 20 minutes. Absorbance was measured at 550 nm. Standard curves were generated for each experiment using reagent hydroxyproline (Sigma H-1637) as a standard. The amount (μg) of hydroxyproline were calculated by comparison to the standard curve.

#### Quantification and statistical analysis.

All results are expressed as mean values ± SEM. The ‘n’ represents biological replicates and can be found in the figure legends. The significance of differences between 2 sample means was determined by unpaired two-tailed student’s t-test (assuming unequal or equal variances as determined by the F-test of equality of variances). All datasets followed a normal distribution and P values less than 0.05 were considered statistically significant. The number of samples to be used was based on the number of experimental paradigms multiplied by the number in each group that is necessary to yield statistically significant results (based on power analysis, to reject the null hypothesis with 80% power (type I error = 0.05).

## Supplementary Material

Supplement 1
