# Supplementary material for: Cell competition drives bronchiolization and pulmonary fibrosis
Source: Res Sq. 2024 Apr 22:rs.3.rs-4177351. Preprint. [Version 1] doi: 10.21203/rs.3.rs-4177351/v1 (PMC11092845; doi:10.21203/rs.3.rs-4177351/v1)
Supplement: Supplement 1 [file NIHPPrs4177351v1-supplement-1.pdf]

Figure S1. Myoepithelial-like cells give rise to basal cells in the lung parenchyma after injury. (A, B) *Nkx2.1^{Flpo};Acta2-Frt-STOP-Frt^{CreERT2};mTmG* mice wer eplace on tamoxifen containing chow for 3 weeks. Following a 3 week washout perioed, mice were infected with H1N1.

At 6 weeks after injury, left lung lobes and trachea were inflation fixed, embedded in paraffin, and sectioned. Coimmunostaining for myoepithelial cell markers Acta2, Krt5, and lineage label GFP on *Nkx2.1^{Flpo};Acta2-Frt-STOP-Frt^{CreERT2};mTmG* trachea (E) and lung (F). (C-D) *Nkx2.1^{Flpo};Acta2-Frt-STOP-Frt^{CreERT2};mTmG* mice were intranasally administered H1N1. At 2 weeks after injury mice were placed on tamoxifen containing chow. At 6 weeks after injury, left lung lobes and trachea were inflation fixed, embedded in paraffin, and sectioned. Coimmunostaining for Krt5, Krt8, and GFP on *Nkx2.1^{Flpo};Acta2-Frt-STOP-Frt^{CreERT2};mTmG* lungs. (E) *Trp63^{DreERT2};Acta2^{CreERT2};RLTG* mice were intranasally administered H1N1 and placed on tamoxifen containing chow at 2 weeks after injury. tdTomato is induced in only *Trp63^{DreERT2}* expressing cells and GFP is induced only when both Trp63^{DreERT2} and *Acta2^{CreERT2}* are expressed

(myoepithelial cells). Coimmunostaining for RPF (tdtomato) and GFP on lungs at 6 weeks after injury. (F) *Sox9^{CreERT2};tdTomato* mice were intranasally administered H1N1 and placed on tamoxifen containing chow at 2 weeks after injury. Coimmunostaining for RFP (tdTomato) and Krt5 on lungs at 6 weeks after injury. Scale bar: 100μm.

Figure S2. Inactivation of Myc inhibits bronchiolization. Mice were placed on tamoxifen chow for 3 weeks and following a 3 week washout period, mice were injured with intratracheal administration of bleomycin. At 6 weeks post injury, left lung lobes were inflation fixed, embedded in paraffin, and sectioned. Spatial transcriptomics was performed on Control and *Sox2^{CreERT2};Myc$^{f/f}$* lungs. Projection of spot clusters onto immunofluorescence image of tissue samples. Spatial gene expression transcripts representing basal-like cells (*Krt5, Krt14, Krt17),* secretory cells (*Bpifa1, Reg3g, Cyp2f2),* goblet cells (*Muc5b),* ciliated cells (*Foxj1),* serous cells (*Scgb3a2, Ltf*)*,* alveolar fibroblasts (*Scube2, Scara5),* AT1 cells *(Hopx, Ager)* and AT2 cells (*Sftpc)* markers were mapped onto spot coordinates. Color scale reflects abundance of indicated transcripts.

Figure S3. Myc promotes bronchiolization and inhibits alveolar regeneration. Mice were placed on tamoxifen chow for 3 weeks and following a 3 week washout period, mice were injured with intratracheal administration of bleomycin and placed on doxycycline containing chow to induce *Myc* overexpression. At 6 weeks post injury, left lung lobes were inflation fixed, embedded in paraffin, and sectioned. Spatial transcriptomics was performed on *Scgb1a1^{CreER};LSL-rtTA;Tet-Myc* lungs. Projection of spot clusters onto immunofluorescence image of tissue samples. Spatial gene expression transcripts representing basal-like cells (*Krt5, Krt14, Krt17),* secretory cells (*Bpifa1, Reg3g, Cyp2f2),* goblet cells (*Muc5b),* ciliated cells (*Foxj1),* serous cells (*Scgb3a2, Ltf*)*,* alveolar fibroblasts (*Scube2, Scara5),* AT1 cells *(Hopx, Ager)* and AT2 cells (*Sftpc)* markers were mapped onto spot coordinates. Color scale reflects abundance of indicated transcripts.

Figure S4. Inhibition of *Yap/Taz* promotes bronchiolization. Mice were placed on tamoxifen chow for 3 weeks and left lung lobes were inflation fixed, embedded in paraffin, and sectioned.

Spatial transcriptomics was performed on *Sox2*^{CreERT2}*;Stk3*$^{f/f}$*;Stk4*$^{f/f}$ and *Sox2*^{CreERT2}*;Yap1*$^{f/f}$*;Wwtr1*$^{f/f}$ lungs. Projection of spot clusters onto immunofluorescence image of tissue samples. Spatial gene expression transcripts representing basal-like cells (*Krt5, Krt14, Krt17),* secretory cells (*Bpifa1, Reg3g, Cyp2f2),* goblet cells (*Muc5b),* ciliated cells (*Foxj1),* serous cells (*Scgb3a2, Ltf*)*,* alveolar fibroblasts (*Scube2, Scara5),* AT1 cells *(Hopx, Ager)* and AT2 cells (*Sftpc)* markers were mapped onto spot coordinates. Color scale reflects abundance of indicated transcripts.

Figure S5. Club cells are actively maintained by cytoplasmic Taz. Left lung lobes were inflation fixed, embedded in paraffin, and sectioned. (A-C) Immunostaining for Sftpc (A-C), Merlin (A, C), and Scgb1a1 (B, C) and immunostaining for phosphorylated-Stk3/4 (D) and phosphorylated-Yap (E) on control non-injured (NI) lungs. (F-K) Mice were placed on tamoxifen chow for 3 weeks and left lung lobes were inflation fixed, embedded in paraffin, and sectioned. (F-G) Immunostaining for Rage, Scgb1a1, and Sftpc on control and *Scgb1a1*^{CreER}*;Nf2*$^{f/f}$ lungs. (H-K) Immunostaining for Krt5, Muc5b, and Scgb1a1 (H-I), beta-tubulin and Scgb1a1 (J), and Acta2 and Scgb1a1 (K) on control (H) and *Sox2*^{CreERT2}*;Yap1*$^{f/f}$*;Wwtr1*$^{f/f}$ (I-K) lungs. (L) Log_2 normalized values for RNA expression for *Agbl4, Foxj1, Scgb3a2, Muc5b, Fn1,* and *Scgb1a1* from NanoString analysis on tamoxifen treated NI control (n=15), *Sox2*^{CreERT2}*;Yap1*$^{f/f}$*;Wwtr1*$^{f/f}$ (n=6), *Sox2*^{CreERT2}*;Yap1*$^{f/f}$(n=4), *Sox2*^{CreERT2}*;Wwtr1*$^{f/f}$(n=4), and *Sox2*^{CreERT2}*;Stk3*$^{f/f}$*;Stk4*$^{f/f}$(n=5). Scale bars: 100μm (A-C), 200μm (D-E, J-K), 250μm (F-G), 500μm (H-I). Student's T test was used to determine significance. *p<0.05, **p<0.01, ***p<0.001, ####p<0.00000001.

Figure S6. Overexpression of a dominant active *Yap*^{S112A} in BESCs is sufficient to drive neo-BCs differentiation. (A) *Sox2*^{CreERT2}*;mTmG* and *Sox2*^{CreERT2}*;Yap1*$^{f/f}$*;mTmG* were placed on tamoxifen containing chow at 8 weeks of age for 3 weeks to inactivate Yap and permanently label all bronchial epithelial cells and their offspring with GFP. After a 3 week wash-out period, mice were intranasally administered H1N1 influenza virus. At 6 weeks post injury, left lung lobes were inflation fixed, embedded in paraffin, and sectioned. (B, E) Coimmunostaining for Krt5 (basal cells)

and Acta2 (myofibroblasts) and (C, F) coimmunostaining for Rage (AT1 cells), GFP (lineage label), Scgb1a1 (Club cells/BASCs), and Sftpc (AT2 cells) on *Sox2^{CreERT2};mTmG* and *Sox2^{CreERT2};Yap1$^{f/f}$;mTmG*. (D) Model demonstrating that Myc sufficient/Hippo active Club cells inhibit basal cells and give rise to alveolar epithelial cells after influenza injury. (G) Model demonstrating that Yap deficient bronchial epithelial cells fail to give rise to basal cells but can give rise to alveolar epithelial cells after influenza injury. (H) Hydroxyproline analysis for soluble collagen on *Cre-* control, *Sox2^{CreERT2};Yap1$^{f/f}$;mTmG*. (I) Survival curve for *Cre-* control, *Sox2^{CreERT2};Yap1$^{f/f}$;mTmG.* (N) Control and *Sox2^{CreERT2};LSL-rtTA;Tet-Yap1^{S112A}-H2BGFP* mice were placed on tamoxifen chow for 3 weeks and following a 3 week washout period, mice were injured with intratracheal administration of bleomycin and placed on doxycycline containing chow to induce *Yap* overexpression. At 5 weeks post injury, left lung lobes were inflation fixed, embedded in paraffin, and sectioned. (J, K) Immunostaining for Krt8 (transitional and bronchial epithelial cells), GFP (*Yap1* overexpressing cells), Krt5 (basal cells) (J), with magnification in (K). (L) Coimmunostaining Myc, p63, and GFP on *Sox2^{CreERT2};LSL-rtTA;Tet-Yap1^{S112A}*. (M) Model demonstrating that Yap overexpressing bronchial epithelial cells basal cells but fail to give rise to alveolar epithelial cells after bleomycin injury. Scale bars: 100μm (B-E), 50μm (H, J) and 25μm (I). Student's T test was used to determine significance. * $p<0.05$.

Figure S7. Yap is required for Myc induced bronchiolization. *Sftpc^{CreERT2};LSL-rtTA;Tet-Myc;Tet-Yap^{S112A}-H2BGFP, Sftpc^{CreERT2};LSL-rtTA;Tet-Myc,* and *Sftpc^{CreERT2};LSL-rtTA;Tet-Yap1^{S112A}-H2BGFP* were placed on tamoxifen containing chow at 8 weeks of age for 3 weeks. After a 3 week washout, mice were intratracheally administered bleomycin and placed on doxycycline containing chow to induce Myc and/or dominant active Yap1^{S112A}. At 6 weeks post injury, left lung lobes were inflation fixed, embedded in paraffin, and sectioned. (A, E, I) Coimmunostaining for myoepithelial like-cell markers (Krt5, Sox9, Myc). (B, F, J) Coimmunostaining for Krt5 and Yap. In the absence of *Tet-Myc*, Yap1^{S112A} does not induce Yap overexpression in AT2 cells. (C, G, K) Coimmunostaining for Krt5 and Muc5b (mucus producing

secretory cells). (D, H, L) Models depicting that both Myc and Yap overexpression in AT2 cells promotes their differentiation into basal cell pods but single overexpression of Myc or Yap in AT2 cells do not give rise to basal cells. Scale bars: 100μm.

Figure S1

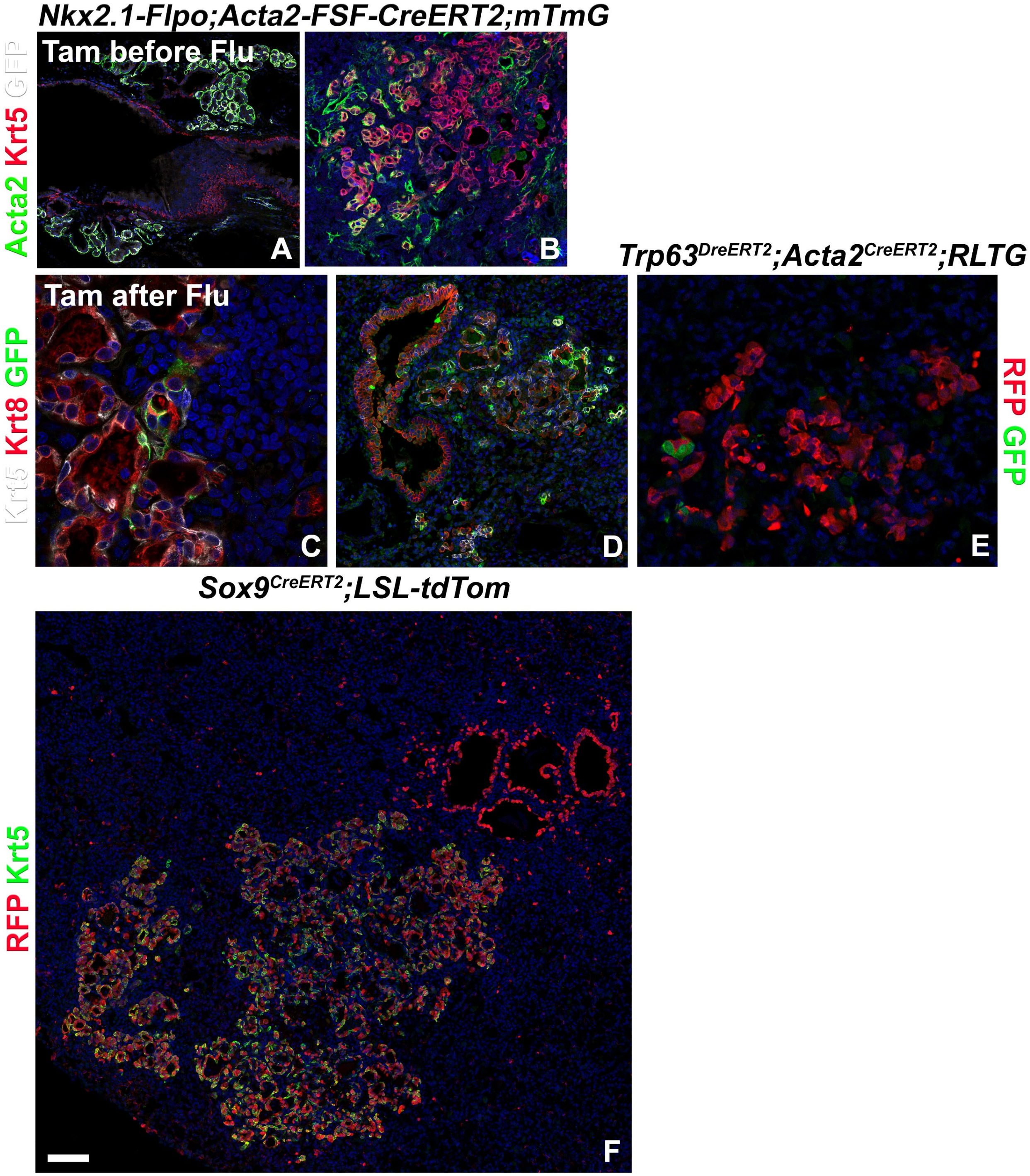

Nkx2.1-Flpo;Acta2-FSF-CreERT2;mTmG
Tam before Flu
Acta2 Krt5 GFP
A
B
Trp63DreERT2;Acta2CreERT2;RLTG
Tam after Flu
Krt5 Krt8 GFP
C
D
RFP GFP
E
Sox9CreERT2;LSL-tdTom
RFP Krt5
F

Figure S2

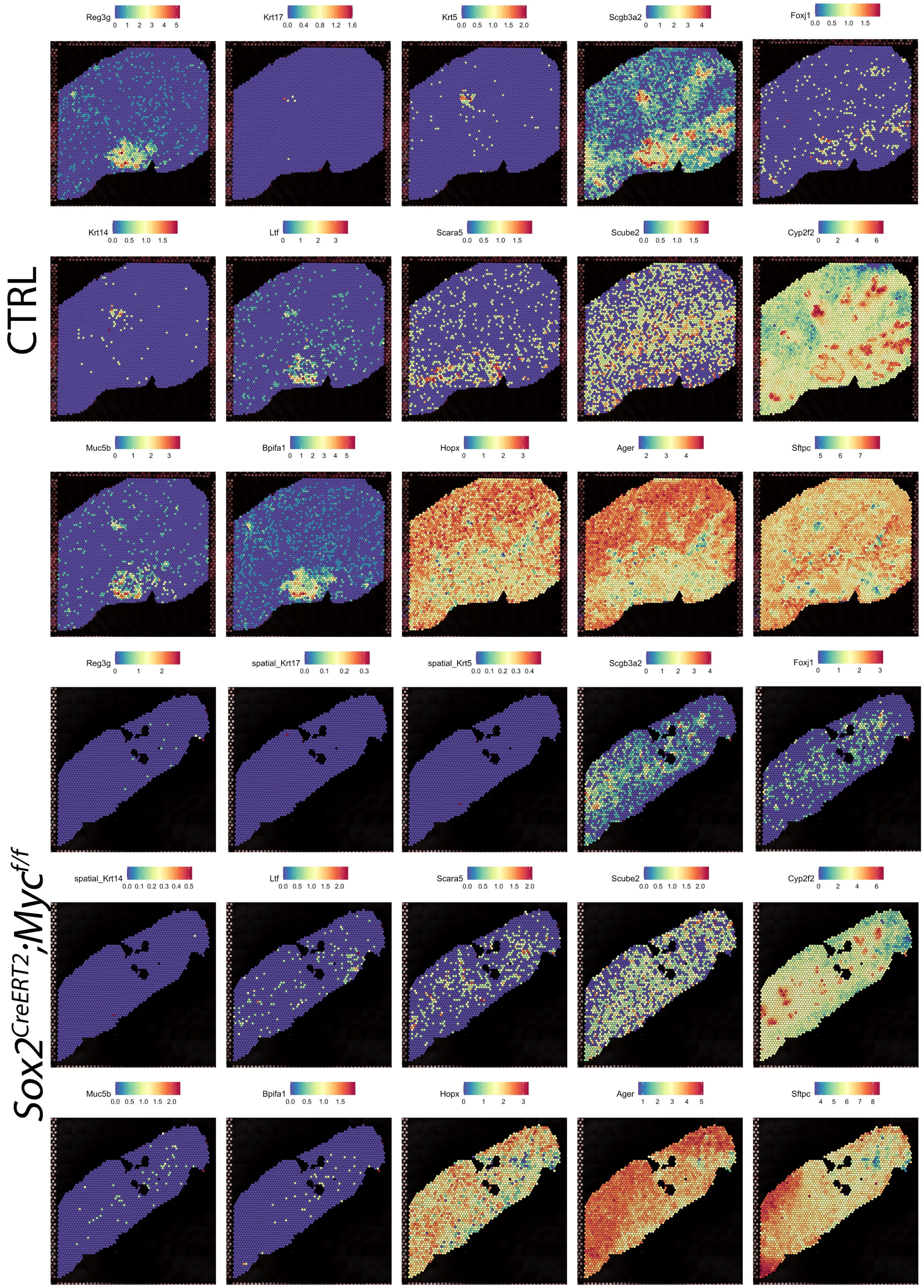
CTRL
Sox2CreERT2;Mycf/f
Reg3g
Krt17
Krt5
Scgb3a2
Foxj1
Krt14
Ltf
Scara5
Scube2
Cyp2f2
Muc5b
Bpifa1
Hopx
Ager
Sftpc
spatial_Krt17
spatial_Krt5
spatial_Krt14

Figure S3

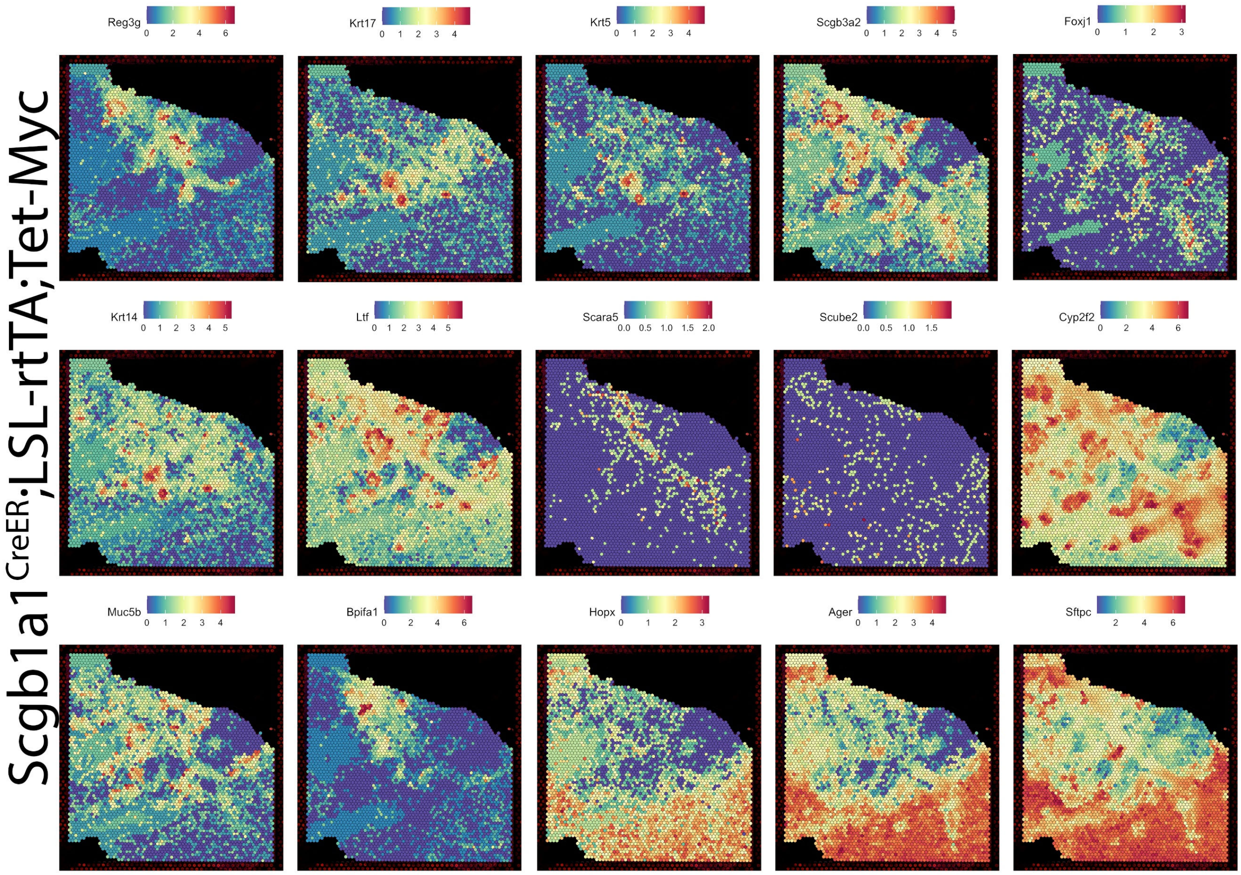
Scgb1a1CreER;LSL-rtTA;Tet-Myc
Reg3g
Krt17
Krt5
Scgb3a2
Foxj1
Krt14
Ltf
Scara5
Scube2
Cyp2f2
Muc5b
Bpifa1
Hopx
Ager
Sftpc

Figure S4

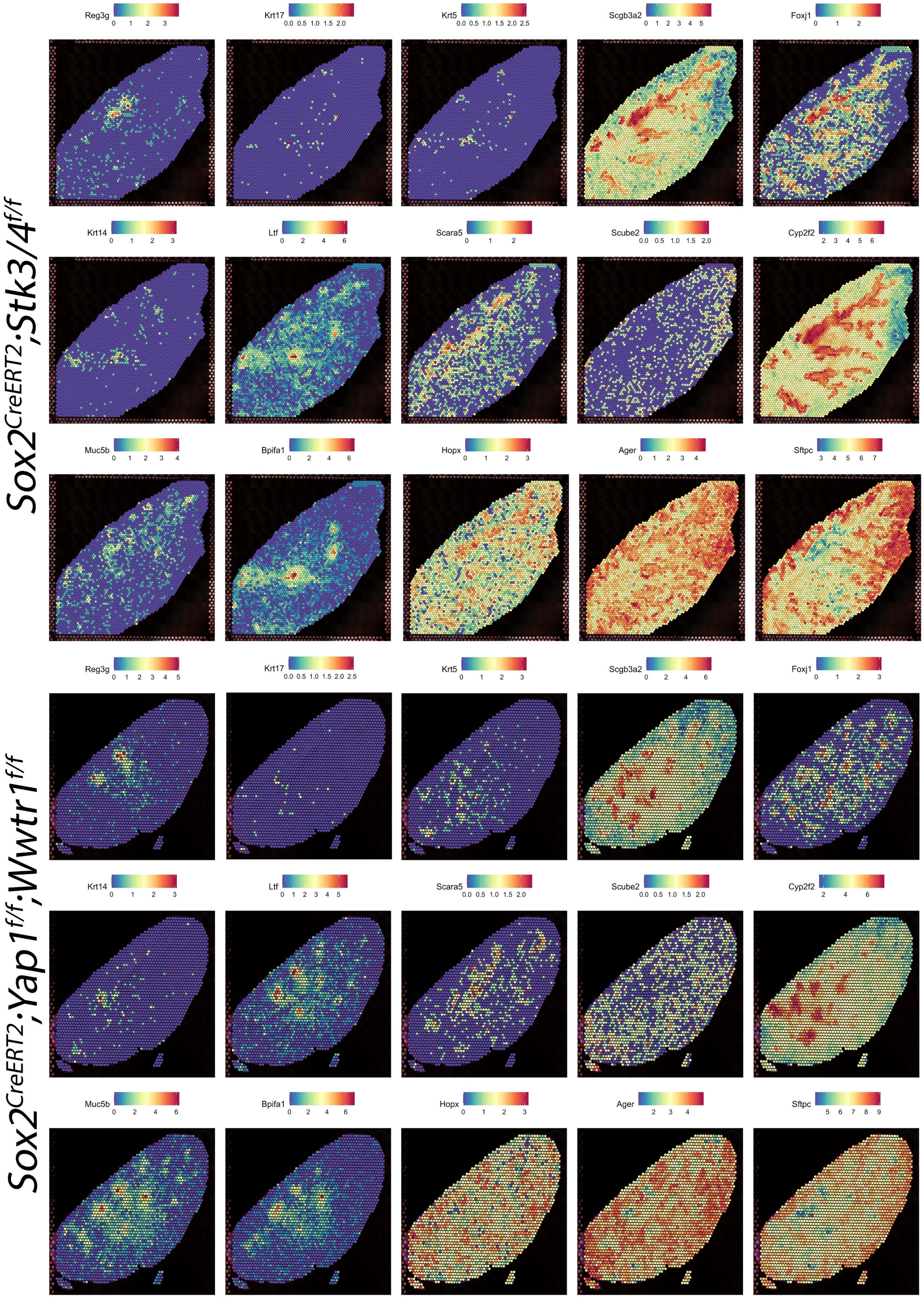
Sox2CreERT2;Stk3/4f/f
Sox2CreERT2;Yap1f/f;Wwtr1f/f
Reg3g
Krt17
Krt5
Scgb3a2
Foxj1
Krt14
Ltf
Scara5
Scube2
Cyp2f2
Muc5b
Bpifa1
Hopx
Ager
Sftpc

Figure S5

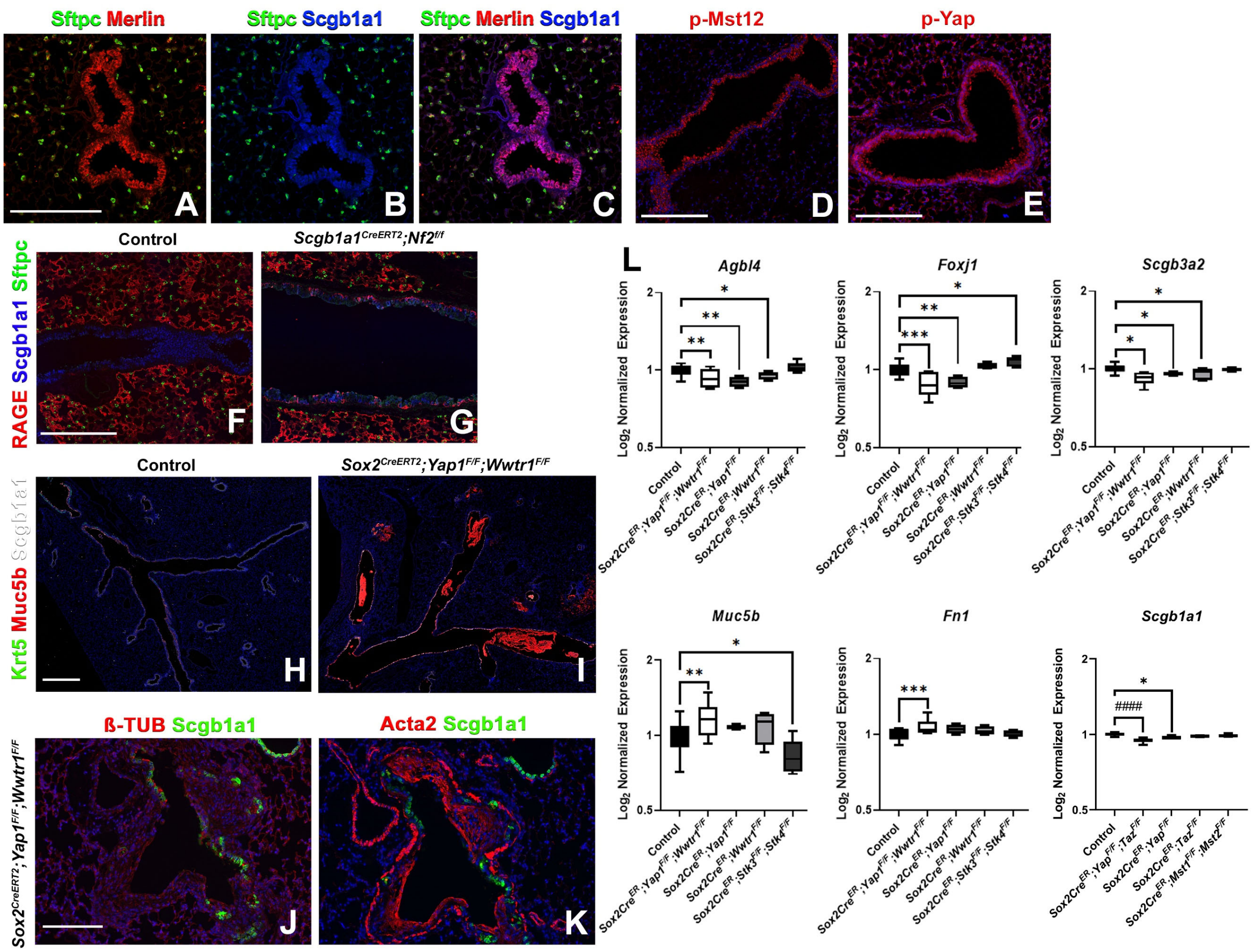

Sftpc Merlin
Sftpc Scgb1a1
Sftpc Merlin Scgb1a1
p-Mst12
p-Yap
A
B
C
D
E
RAGE Scgb1a1 Sftpc
Control
Scgb1a1CreERT2;Nf2f/f
F
G
Krt5 Muc5b Scgb1a1
Control
Sox2CreERT2;Yap1F/F;Wwtr1F/F
H
I
Sox2CreERT2;Yap1F/F;Wwtr1F/F
ß-TUB Scgb1a1
Acta2 Scgb1a1
J
K
L
Agbl4
Foxj1
Scgb3a2
Muc5b
Fn1
Scgb1a1
Log2 Normalized Expression
Control
Sox2CreER;Yap1F/F;Wwtr1F/F
Sox2CreER;Yap1F/F
Sox2CreER;Wwtr1F/F
Sox2CreER;Stk3F/F;Stk4F/F
Sox2CreER;YapF/F;TazF/F
Sox2CreER;YapF/F
Sox2CreER;TazF/F
Sox2CreER;Mst1F/F;Mst2F/F
####

Figure S6

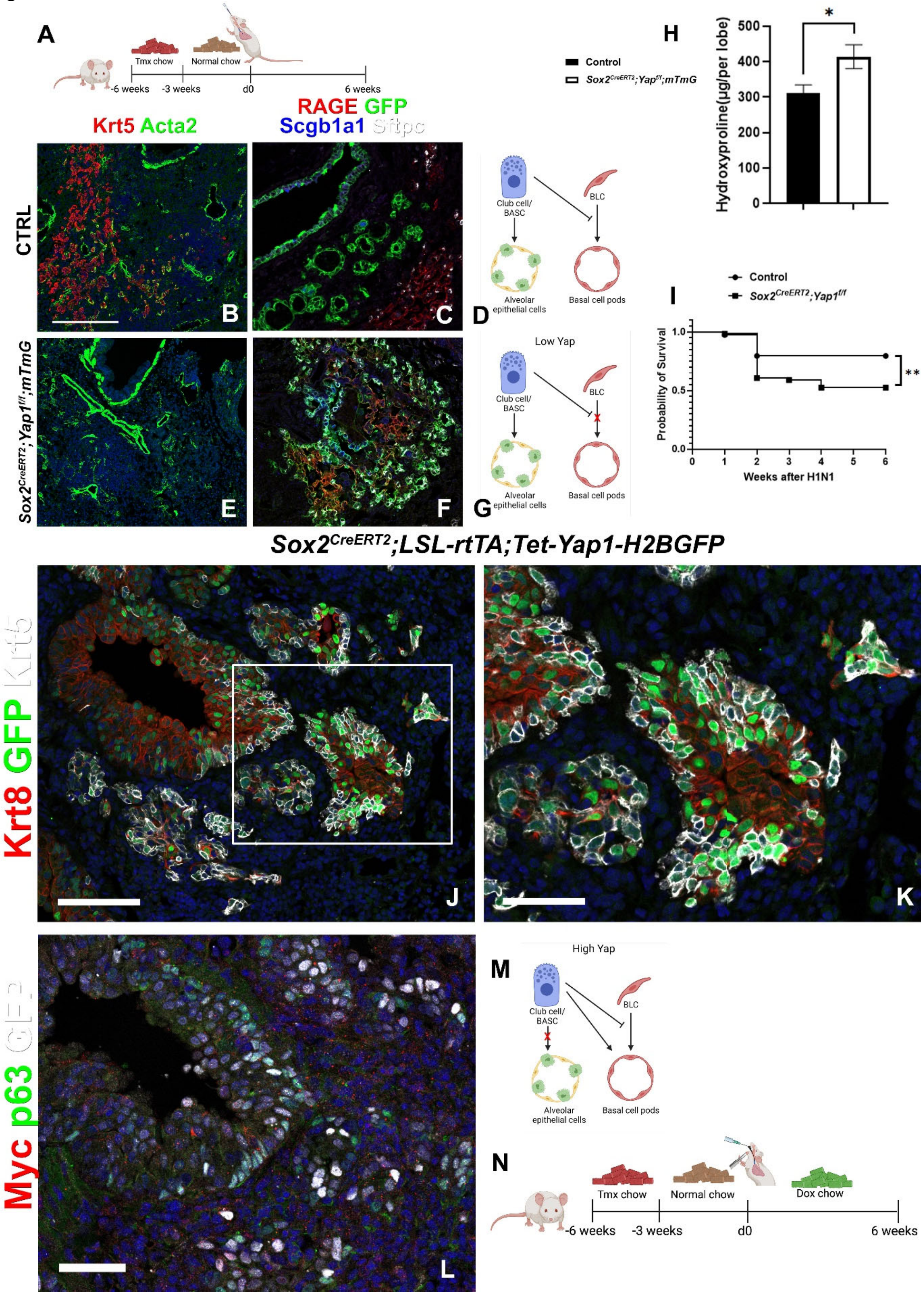

A
Tmx chow
Normal chow
-6 weeks
-3 weeks
d0
6 weeks
Krt5 Acta2
RAGE GFP
Scgb1a1 Sftpc
CTRL
Sox2CreERT2;Yap1f/f;mTmG
B
C
E
F
Club cell/BASC
BLC
Alveolar epithelial cells
Basal cell pods
D
Low Yap
G
H
Control
Sox2CreERT2;Yapf/f;mTmG
Hydroxyproline(μg/per lobe)
500
400
300
200
100
0
*
I
Control
Sox2CreERT2;Yap1f/f
Probability of Survival
1.0
0.5
0.0
0
1
2
3
4
5
6
Weeks after H1N1
**
Sox2CreERT2;LSL-rtTA;Tet-Yap1-H2BGFP
Krt8 GFP Krt5
J
K
Myc p63 GFP
L
M
High Yap
N
Dox chow

Figure S7

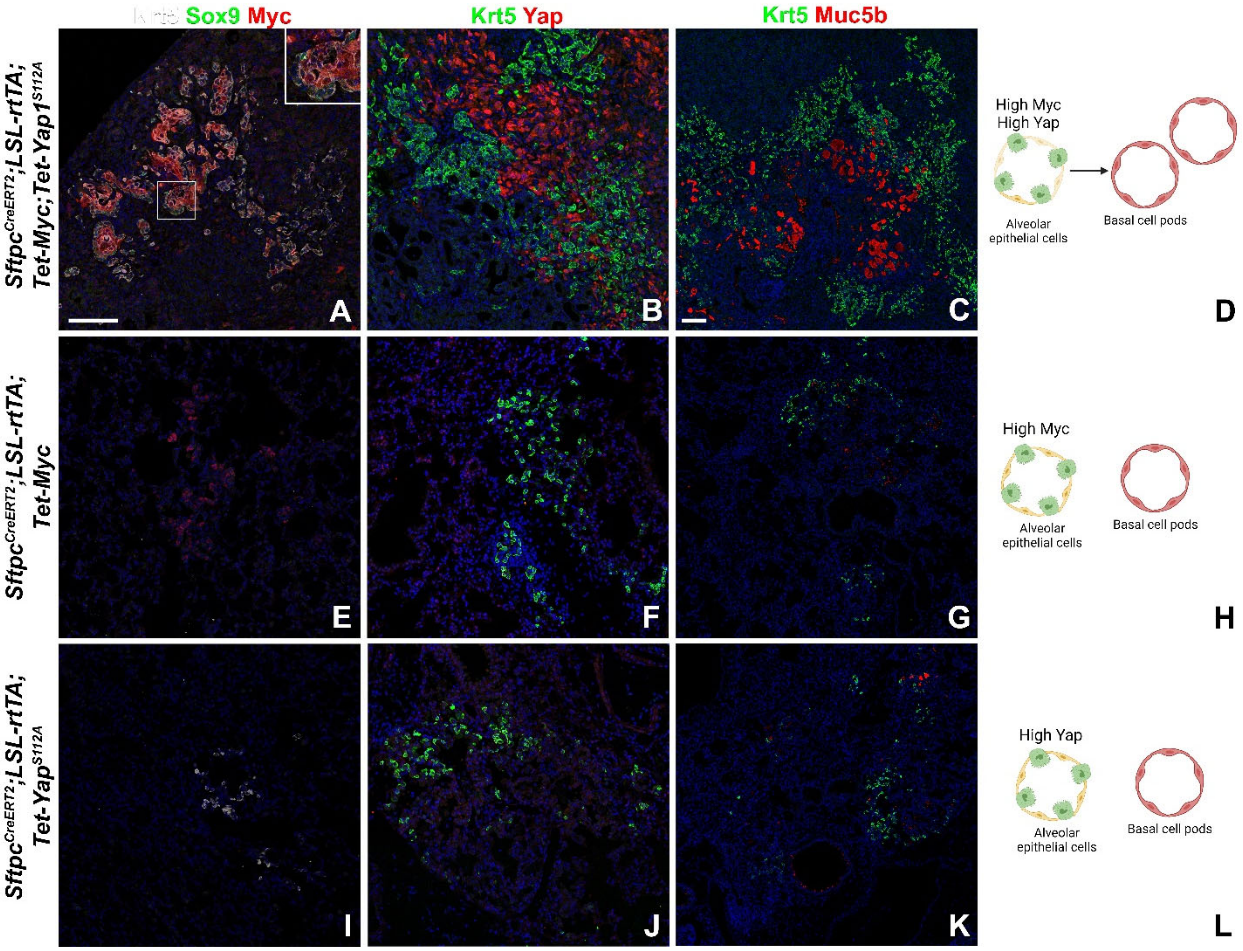

Sox9 Myc
Krt5 Yap
Krt5 Muc5b
Sftpc^CreERT2;LSL-rtTA; Tet-Myc;Tet-Yap1^S112A
Sftpc^CreERT2;LSL-rtTA; Tet-Myc
Sftpc^CreERT2;LSL-rtTA; Tet-Yap^S112A
High Myc
High Yap
Alveolar epithelial cells
Basal cell pods
A
B
C
D
E
F
G
H
I
J
K
L
